# Precise Cerebral Vascular Atlas in Stereotaxic Coordinates of Whole Mouse Brain

**DOI:** 10.3389/fnana.2017.00128

**Published:** 2017-12-19

**Authors:** Benyi Xiong, Anan Li, Yang Lou, Shangbin Chen, Ben Long, Jie Peng, Zhongqin Yang, Tonghui Xu, Xiaoquan Yang, Xiangning Li, Tao Jiang, Qingming Luo, Hui Gong

**Affiliations:** ^1^Britton Chance Center for Biomedical Photonics, Wuhan National Laboratory for Optoelectronics-Huazhong University of Science and Technology, Wuhan, China; ^2^MoE Key Laboratory for Biomedical Photonics, Collaborative Innovation Center for Biomedical Engineering, School of Engineering Sciences, Huazhong University of Science and Technology, Wuhan, China

**Keywords:** whole mouse brain, three-dimensional reconstruction, fine vascular atlas, vascular distributing patterns, microvessels, quantitative calculation

## Abstract

Understanding amazingly complex brain functions and pathologies requires a complete cerebral vascular atlas in stereotaxic coordinates. Making a precise atlas for cerebral arteries and veins has been a century-old objective in neuroscience and neuropathology. Using micro-optical sectioning tomography (MOST) with a modified Nissl staining method, we acquired five mouse brain data sets containing arteries, veins, and microvessels. Based on the brain-wide vascular spatial structures and brain regions indicated by cytoarchitecture in one and the same mouse brain, we reconstructed and annotated the vascular system atlas of both arteries and veins of the whole mouse brain for the first time. The distributing patterns of the vascular system within the brain regions were acquired and our results show that the patterns of individual vessels are different from each other. Reconstruction and statistical analysis of the microvascular network, including derivation of quantitative vascular densities, indicate significant differences mainly in vessels with diameters less than 8 μm and large than 20 μm across different brain regions. Our precise cerebral vascular atlas provides an important resource and approach for quantitative studies of brain functions and diseases.

## Introduction

The mammalian brain is the organ that consumes the most energy. The oxygen and nutrients used to maintain the normal function of the neurons and glial cells in brain tissues are supplied continuously by the bloodstream (Iadecola, 2004; Attwell et al., 2010; Hirsch et al., 2012). A fine and complete vascular system of arteries, veins and capillaries provides the structural basis for ensuring the supply from the bloodstream (Zlokovic, 2005; Meyer et al., 2008; Hirsch et al., 2012; Zhang et al., 2012). Alterations of the vascular structure can be found in brain pathologies, such as stroke, Alzheimer's, and tumors (Meyer et al., 2008; Blinder et al., 2010; Hirsch et al., 2012; Xie et al., 2012). The arteries transport the blood to the different brain regions while veins collect the blood. Both the arteries and veins are very important to the brain as the consequences of occlusion of arteries and veins are well known (Sekhar et al., 2002; Blinder et al., 2010). Therefore, the structures of the arterial and venous vascular system, the distributing patterns of the vascular system within the brain tissues and the detailed information of microvessels form a basis for understanding brain function and pathology (Hirsch et al., 2012). However, our knowledge of the cerebral vascular system remains remarkably incomplete, especially in veins.

Recently, great progress has been made in many techniques used to image whole brain vascular structures. For example, μCT combined with vascular casting (Heinzer et al., 2006, 2008), MOST (Li et al., 2010) using Indian-Ink perfusion (Xue et al., 2014) and Nissl staining (Wu et al., 2014, 2016; Yuan et al., 2015), KESM combined with Indian-Ink perfusion (Mayerich et al., 2011; Ryang et al., 2011; Pesavento et al., 2017), and Nissl staining (Mayerich D. et al., 2008; Mayerich D.M. et al., 2008), serial two-photon tomography (STP) combined with fluorescence labeling (Amato et al., 2016), can be used to acquire high-resolution mouse cerebral vascular datasets in large areas. The vascular casting and Indian-Ink perfusion method can produce high quality and resolution vascular images, though the cytoarchitecture is not included in the reported and public data. Some fantastic reconstructed and statistical results of the vessels have been acquired in the cerebral cortex (Tsai et al., 2009; Blinder et al., 2013; Uludag and Blinder, 2017) and some useful analysis methods and parameters were proposed (Cassot et al., 2006; Tsai et al., 2009; Lorthois et al., 2014; Wu et al., 2014). However, there have been no reports of the fine vascular system of both the arteries and veins in one and the same mouse brain. Studies of the cerebral vascular system have mainly focused on the arteries (Dorr et al., 2007; Scremin, 2012), and studies of veins remain very scarce and have been limited to the level of the primary veins in the pial surface and inside the brain (Dorr et al., 2007; Scremin, 2012). The precise relation between blood vessels and brain regions, reflecting the physiology and pathology of brain function directly and accurately, has remained largely unknown. Thus, a comprehensive, systematic study of the distributing patterns of each artery and vein within brain regions in the same mouse brain is needed. In previous studies, brain regions were only considered to orientate the vessels, and provided incomplete location information between vessels and brain regions (Scremin, 1995, 2012; Dorr et al., 2007). Functional interaction between the cerebral vessels and brain tissues occurs in the microvessels, where the neurovascular unit is generated (Zhang et al., 2012). But the statistical results for microvessels in 3D have mainly been acquired in cortical areas (Tsai et al., 2009; Blinder et al., 2013; Wu et al., 2014, 2016) and there is no detailed information of 3D microvessels across different brain regions. Acquiring the fine and complete vascular system of arteries, veins, microvessels, and the distributing patterns between the vascular system and brain regions in the whole mouse brain, remains a core urgent need of neuroscience.

To construct a standard mouse cerebral vascular system atlas to benefit studies of cerebral vascular diseases and cerebral vascular development, we acquired datasets containing both the vessels and cytoarchitecture of the whole mouse brain with a voxel resolution of 1 μm using modified Nissl staining and MOST imaging. We reconstructed the precise vascular system of both the arteries and veins, and accurately located and uniformly annotated the vascular system based on the cytoarchitecture, which was regarded as the gold standard for anatomical localization. Then, we constructed an atlas of the precise vascular system of both arteries and veins, followed by a quantitative study of the distributing patterns between cerebral vessels and brain regions in the whole brain, using the data with voxel size of 5 × 5 × 5 μm^3^. And we quantitatively counted the branch numbers of all the arteries and veins. Subsequently, we analyzed the microvessels in the primary somatosensory cortex, barrel field (S1BF), superior colliculus (SC), hippocampus (HIP), thalamus (TH), and hypothalamus (HY), using the data with voxel size of 0.35 × 0.35 × 1 μm^3^. And we obtained quantitative results for the fractional vascular volume (Fv) and normalized vascular length (Nl).

## Materials and methods

### Sample preparation

The image datasets in this study were obtained from five 8-week-old, male C57BL/6 mice with Nissl staining (Wu et al., 2014). First, the mice were anesthetized using a 1% solution of sodium pentobarbital (90 mg/kg) and perfused intracardially with 0.01M phosphate-buffered saline (PBS) and 4% paraformaldehyde at a pressure of 38 mmHg. After perfusion, the brains were removed from the skull and post-fixed. Subsequently, the brains were washed with PBS and stained with thionine solution. Then a graded series of ethanol and acetone solutions were used to dehydrate the brains. Finally, the brains were immersed in a graded series of Spurr resin solutions. The total sample preparation time was nearly 25 days. Detailed information about sample preparation can be found in our previous work (Wu et al., 2014). All the animal experiments were performed according to the procedures approved by the Institutional Animal Ethics Committee of Huazhong University of Science and Technology.

### Imaging

The embedded whole-brain specimens were imaged with MOST (Li et al., 2010), which performed simultaneous thin sectioning and imaging while simultaneously recording the image coordinates for automatic aligning of the images. The high-resolution images had a voxel size of 0.35 × 0.35 × 1 μm and were all saved at a depth of 8 bits. For each specimen, uninterrupted imaging was performed for 7 days, and the size of the uncompressed image dataset exceeded 2.4 terabytes with more than 11,000 coronal sections.

### Image preprocessing and spatial orientation correction

Due to the uneven staining and illumination, the original images were preprocessed to correct for brightness and to remove noise using a customized preprocessing program (Ding et al., 2013). Then, the spatial orientation was corrected. The preprocessed raw data is shown in Supplementary Figure 1.

The transform pattern between and the original orientation and the standard orientation was obtained by 3D transformation using Amira software (Visage Imaging, San Diego, California). First, the whole mouse brain with a voxel size of 5 × 5 × 5 μm^3^ was visualized, and then the 3D mouse brain was manually transformed to the standard orientation such that the dorsal part was horizontal. Ultimately, we obtained a 4 × 4 transformation matrix T (Gong et al., 2013).

(1) $${\text{A}}^{*}\text{T}=\text{B},\text{T}=\left(\begin{array}{cc} \begin{array}{c} S_{11}\\ S_{21}\\ S_{31} \end{array} & \begin{array}{ccc} S_{12} & S_{13} & 0\\ S_{22} & S_{23} & 0\\ S_{32} & S_{33} & 0 \end{array}\\ t_{11} & \begin{array}{ccc} t_{22} & t_{33} & 1 \end{array} \end{array}\right)$$

A is the original image stack and B is the transformed image stack in 3D. S is the rotation factor, and t is the translation factor in 3 directions. And the gray value of all points in B were acquired with the corresponding points in A through an inverse matrix of T with a customized MATLAB (MathWorks, Natick, MA) program.

### Arteries and veins tracing

#### Accurate binarization of brain contour

The arteries and veins in the whole brain were reconstructed using the datasets with voxel size of 5 × 5 × 5 μm^3^. We down-sampled the data for segmentation of arteries and veins for two reasons: (1) The size of the raw image dataset of whole brain exceed 2,400 GB with more than 11,000 coronal sections (resolution with 0.35 × 0.35 × 1 μm^3^). The existing computer memory and image processing software can not directly deal with such a large amount of data. (2) The arteries, veins and the capillaries are highly interconnected to form a complex network in the whole mouse brain. We can't distinguish and separate the arteries and veins from the complex network.

To obtain accurate brain contours from the dataset, a 3 × 3 sized median filter was first applied to remove noise. Then, the threshold was obtained using the *graythresh* (i.e., Otsu method; Otsu, 1975) function in MATLAB. Subsequently, all sections were binarized using the threshold. The holes were filled, some isolated points were removed via morphological operations (using the *imfill, imclose*, and *imopen* functions in MATLAB), and some noise on a large scale was removed by connected component analysis (He et al., 2014) (using the *bwlabeln* and *regionprops* functions in MATLAB). All processes were accomplished with a customized MATLAB program. Finally, some manual editing was performed for the binarized sections, which were overlaid on the original dataset with the filament editor in Amira software, to get the accurate brain contours.

#### Tracing of pial surface vessels

**Figure 1:**
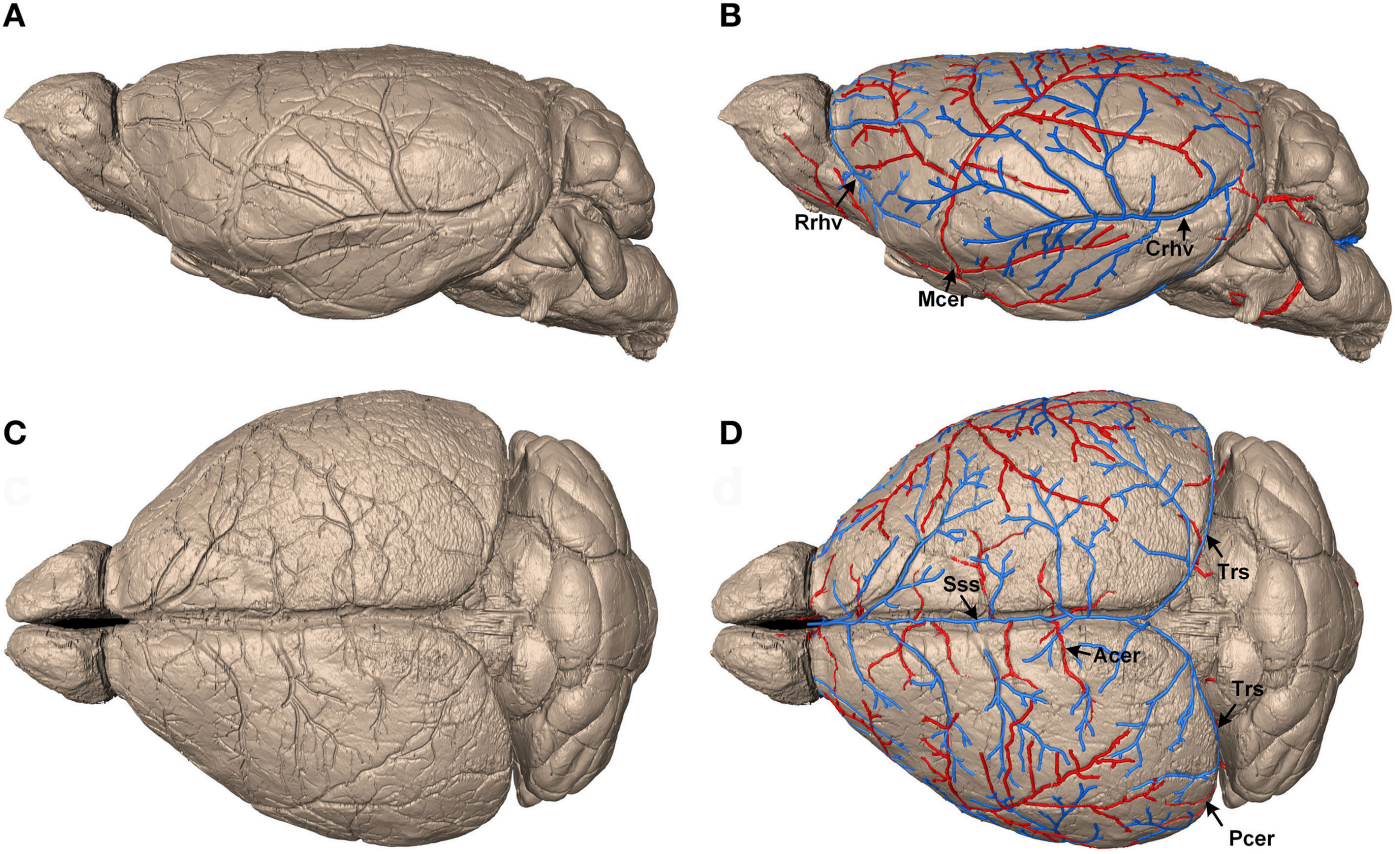
Identification and reconstruction of pial surface vessels. **(A,C)** Reconstructed 3D mouse brain surface with surface rendering on sagittal and horizontal views, respectively (data resolution is 5 × 5 × 5 μm^3^). **(B,D)** Tracing results of the pial surface arteries and veins. Arteries, including Mcer, Acer, and Pcer, are presented in red, whereas veins, including Sss, Rrhv, Crhv, and Trs, are in blue. Mcer, Middle cerebral artery; Acer, Anterior cerebral artery; Pcer, Posterior cerebral artery; Sss, Superior sagittal sinus; Rrhv, Rostral rhinal vein; Crhv, Caudal rhinal vein; Trs, Transverse sinuses.

Different sized dents of the pial surface vessels were presented in serial coronal sections in a continuous manner (Supplementary Figure 2). To trace the pial surface vessels in the mouse brain with voxel size of 5 × 5 × 5 μm^3^ (Figure 1), a morphological closing operation (*imclose* functions in MATLAB) was first implemented to fill the dents in the brain contour. The model used for closing was a circle with a radius of 30 pixels. Next, the closed images were subtracted by the original images to extract the continuous dents of the surface vessels. Then, the extracted dents were traced in 3D with NeuronStudio software (Wearne et al., 2005; Rodriguez et al., 2009), and the traced results were saved in .swc (Cannon et al., 1998) format. Ultimately, the vectorized results were edited manually with flNeuronTool (Ming et al., 2013) to correct for discontinuous and redundant vascular branches.

#### Tracing of connected penetrating vessels

Tracing cortical penetrating vessels and distinguishing them as arteries or veins required connecting paths between penetrating vessels and the pial surface vessels (Supplementary Figure 3). Each penetrating vessel within the data with voxel size of 5 × 5 × 5 μm^3^ was semi-automatically segmented in 3D via the region growing method using the filament editor in Amira software and saved as binarized images.

To semi-automatically obtain the binarized results for the penetrating vessels, appropriate thresholds must be acquired. The threshold of each vessel was set manually to be the minimum value (near the value that 20 larger than the mean value of the image) that would obtain as many vascular branches as possible without segmenting background areas, which could be visualized both in 2D and 3D in real time using the Amira software during the segmentation. Subsequently, the seed point of each penetrating vessel was set manually to start the segmentation in 3D. Finally, the segmented binarized results were manually checked and edited to add the missed branches.

We have quantitatively tested that the minimum threshold was about 20 larger than the average gray value of the image within a represented data with size of 585 × 500 × 395 μm^3^ (voxel size of 5 × 5 × 5 μm^3^) as shown in Supplementary Figure 4. We manually segmented all the connected branches of one vessel slice by slice as the ground truth (Supplementary Figure 4d). Then we used different thresholds to segment the same vessel, and all the pixels were used to acquire the F1 score, Recall, Precision, and ROC curves. We defined the binarized pixels with gray value of 255 as the positive results, and the pixels with gray value of 0 as the negative results. The F1 score result showed the suitable threshold was 136 (The mean value of each slice was about 117), and the threshold resulted the Recall and Precision were all large than 98%. The ROC cove showed the suitable threshold was 135 (Recall, Precision > 97%). The results also showed the thresholds within range between 134 and 139, would result the Recall and Precision all large than 94%. Then the thresholds between 134 and 139 were tested manually, the 136 was the minimum threshold as a part of background points were segmented as vessels with thresholds of 135 (Supplementary Figures 4e,f).

### Segmentation of main brain regions

**Figure 2:**
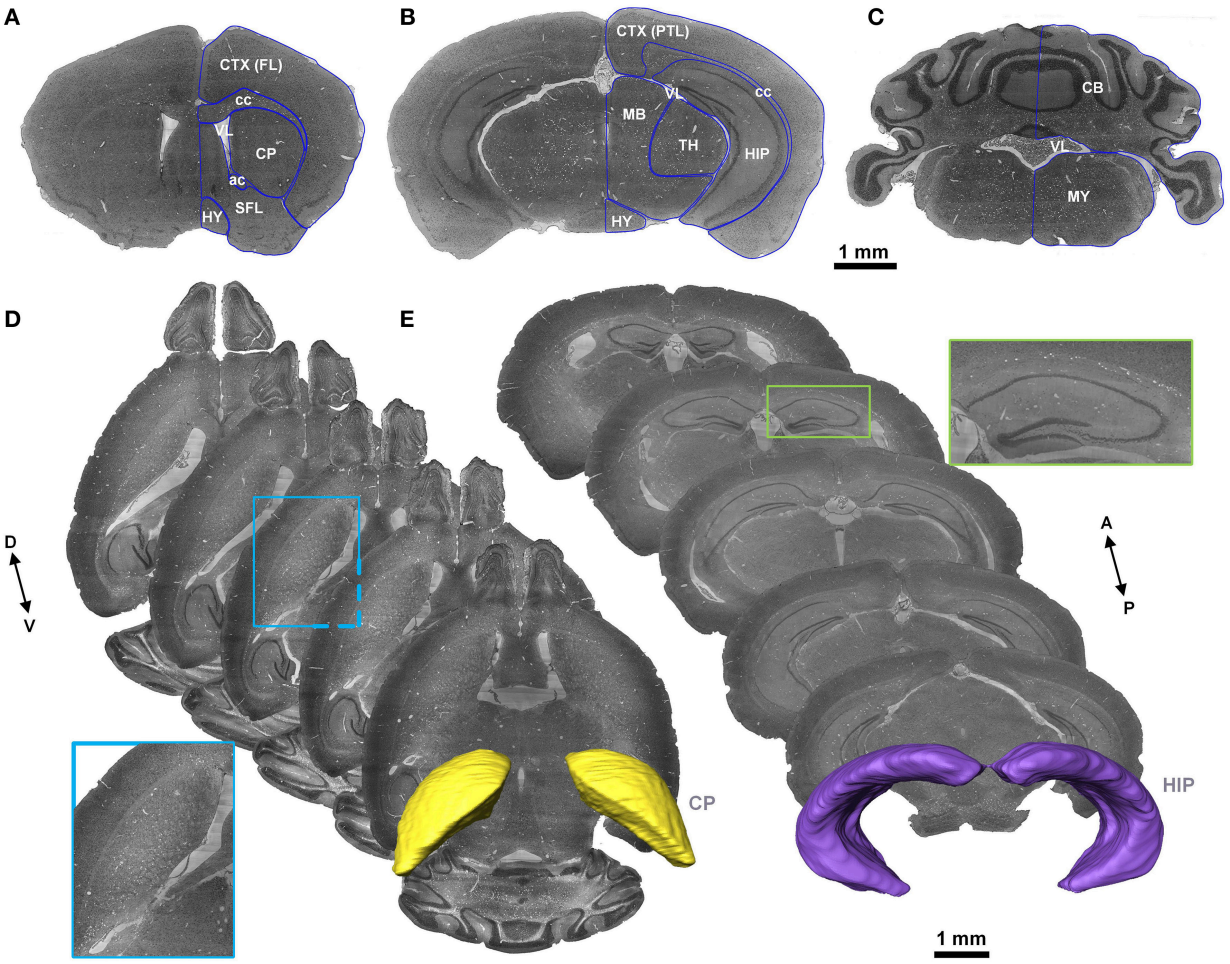
Segmentation of the brain regions. **(A–C)** The representative coronal sections in the same mouse brain. The blue lines represent the contour of the brain regions segmented manually. **(D,E)** Horizontal sections with a distance of 300 μm to present contour indicated by cytoarchitecture of the CP and coronal sections with a distance of 500 μm to present contour indicated by cytoarchitecture of the HIP are presented. The images in the blue and green boxes are local, enlarged views of the CP and HIP, respectively. The 3D structures in yellow and violet are the constructed contours of the CP and HIP, respectively. A, anterior; P, posterior; D, dorsal; V, ventral. A list of abbreviations and the full names of the brain regions is presented in Supplementary Table 1.

Cytoarchitecture with Nissl staining is the basic characteristic that has been used to label brain regions and the nucleus in both the Allen Reference Atlas (Dong, 2011) and the Franklin and Paxinos Mouse Atlas (Paxinos and Franklin, 2012). With reference to the Allen Reference Atlas, the Franklin and Paxinos Mouse Atlas, and the MICe mouse atlas (Dorr et al., 2008), three skilled persons segmented images (voxel size of 5 × 5 × 5 μm^3^) slice by slice with the filament editor in Amira software and then performed back-to-back amendment and validation manually in any of the coronal, sagittal or horizontal sections. The typical segmented brain region contour in 2D sections and 3D space were shown in Figure 2.

### Annotation of arteries and veins

The same with traditional annotating rules, the main annotating rule used here for the mouse cerebral vascular system was “orientation-brain region-artery/vein,” as indicated in the names in black (Supplementary Figure 5). If one position was not sufficient to distinguish the vessels, two or three were used: orientation-orientation-brain region-artery/vein, as indicated in the names in blue. If some branches that connect to the same vessel at the same level were distinguishable with orientation, such as some branches in the pial surface and some third level branches inside the brain, we annotated them “orientation-branch of-name of connected vessel,” as indicated in the names in purple.

### Microvessels tracing and vascular density calculation

To obtain the microvessels in the brain regions, the datasets (voxel size of 0.35 × 0.35 × 1 μm^3^), with a unified size of 400 × 400 × 400 μm^3^ were binarized based on the Otsu method (Otsu, 1975; Wu et al., 2014). The binarized vessels were then traced with the skeletonization method in Amira software. The center lines of the vascular network were acquired via Amira, and a radius-adjustable sphere (Peng et al., 2010) was used to estimate the diameters and the results were saved as .swc format.

We assessed the tracing method both qualitatively (Supplementary Figures 6a–c) and quantitatively (Supplementary Figures 6e–i). Qualitative results in 2D and 3D both showed the diameter and centerline matched well with the raw data. For quantitative assessment, recall (R), precision (P), and diameter ratio (D) were used to estimate the traced results with the ground truth labeled manually, according to a reported method (Wu et al., 2014). Images were extracted every 70 μm, and the traced result and ground truth (segmented manually) were put together. We defined B as the overlapped regions, B1 as the total number of ground truth regions, B2 as the total number of traced regions, A1 and A2 as the areas of the traced and manually labeled regions, So R = B/B1, P = B/B2, D = $\sqrt{A1/A2}$ (Wu et al., 2014). The recall reached 95.7%, while the precision was 94.4%. The diameter ratio was 96.7%. The detailed estimated results was shown in Supplementary Figure 6.

The parameters used for quantitative statistics of the vascular network were fractional vascular volume (Fv) and normalized vascular length (Nl). Firstly, the vessels were separated into 3 groups according to diameter: D > 20 μm; 8 < D ≤ 20 μm; and D ≤ 8 μm. Then, the Fv and Nl were calculated with a MATLAB toolbox, and the results were revised according to the estimated linear shrinkage of 25.9 ± 1.6% (Wu et al., 2014).

### Distributing patterns between vessels and brain regions

To obtain the distribution pattern of arteries and veins within different brain regions, each artery and vein, together with the connected penetrating branches, was first designated with a separate gray value during segmentation, while the 19 brain regions were labeled with different gray values. The segmented results of the vessels and brain regions were saved as separate image stacks with different gray values (voxel size of 5 × 5 × 5 μm^3^). However, both the vessels and brain regions were acquired from the same dataset; thus, their coordinates were matched, and the distribution in different brain regions of each artery and vein could be calculated with the volume of all vascular branches in 3D. The volume of the vessels was calculated by adding together the voxels in in the 3D binarized image stack. The radii of the pial surficial vessels were missing and thus the artery was set to 150 μm and the veins were set to 250 μm uniformly according to the reported mean value (Müller et al., 2008). The calculations and mean distribution results for 5 mouse brains were performed using a customized MATLAB program.

## Results

### Continuous vascular network reconstruction

To construct the precise arterial and venous vascular system in the whole mouse brain, we acquired serial datasets in five 8-week-old male C57BL/6 mice with a voxel resolution of 1 μm using modified Nissl staining and MOST imaging. We traced the pial surface arteries and veins. First, the brain contours of the whole mouse brain were segmented within serial coronal sections (Supplementary Figure 2) based on cytoarchitecture, using the Otsu method (section Materials and Methods), and the 3D brain surface was visualized with surface rendering using Amira (Visage Imaging, San Diego, California) software (Figures 1A,C). This high-quality imaging technology allowed the continuous dents of pial surface vessels to be clearly distinguished on the reconstructed brain surface. Then, based on these dents, we semi-automatically traced the mouse pial surface vessels (see the section Materials and Methods). Finally, arteries, including the middle cerebral artery (Mcer) and pial branches of the anterior cerebral artery (Acer) and posterior cerebral artery (Pcer), and veins, including the superior sagittal sinus (Sss), rostral rhinal vein (Rrhv), caudal rhinal vein (Crhv), and transverse sinuses (Trs) were acquired (Figures 1B,D). The diameter of the smallest terminal branches of the pial surface vessels was as small as 25–30 μm.

To acquire the structures of the cerebral vascular system of the whole mouse brain and to distinguish arteries and veins, we continuously traced the penetrating vessels in large areas using the regional growing method (section Materials and Methods), along the connecting paths with the identified arteries and veins in the pial surface. The fine vascular connecting paths from the pial surface to the deep-brain across the whole brain were acquired, and the diameters of the arterial terminal branches were as small as 8.3 ± 2.6 μm, while the venous terminal branches were as small as 8.7 ± 3.7 μm. To provide a better understanding of the connecting path, Supplementary Figure 3 showed the path between Mcer and the anterior striate artery (Astr) in an individual mouse brain as an example, as a 2D section and a 3D volume rendering. The existing arterial system was fine to about 20 μm (Ghanavati et al., 2014). Here, we reconstructed the 3D fine vascular structures of both the arteries and veins in one and the same mouse brain to make the precise vascular atlas.

### Cytoarchitecture-based, accurate vascular localization, and annotation

We accurately located the cerebral vascular system in the whole mouse brain. Cytoarchitecture is regarded as the gold standard in brain regions labeling (Dong, 2011; Paxinos and Franklin, 2012). Datasets acquired with modified Nissl staining and serial high resolution imaging, provide both vascular structures and cytoarchitecture. Because the cerebral vascular structures and brain regions were imaged at the same time and in the same mouse brain, no more registration was needed. We manually segmented 19 main brain regions (Figure 2, Supplementary Table 1) in each of 5 whole mouse brains (section Materials and Methods) referencing the Allen Reference Atlas (Dong, 2011), the Franklin and Paxinos Mouse Atlas (Paxinos and Franklin, 2012), and the MICe (Mouse Imaging Center) mouse atlas (Dorr et al., 2008). Subsequently, we accurately located the traced vessels in 3D, based on the segmentated brain regions.

**Figure 3:**
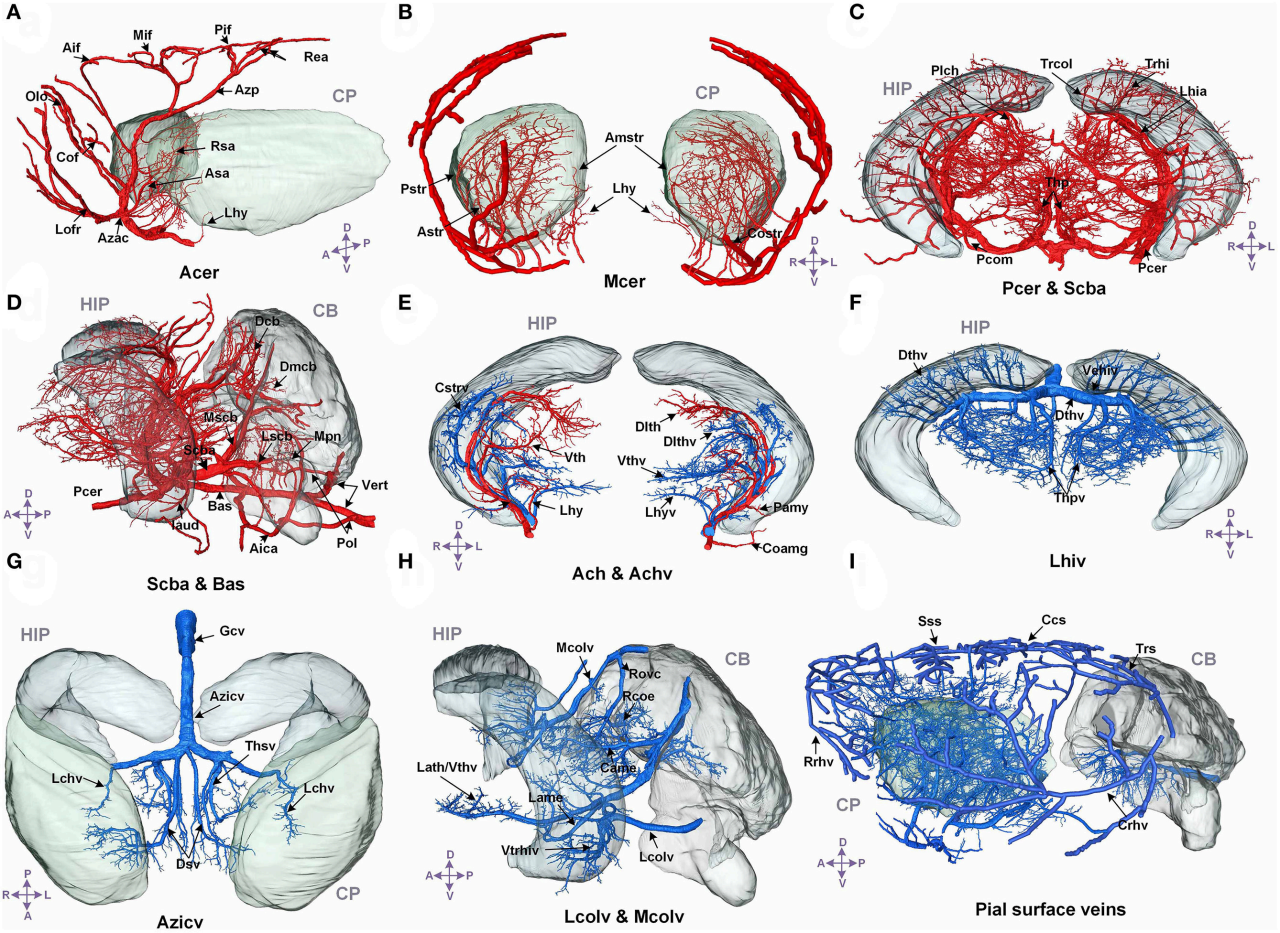
The structure, distribution and labeling of the whole brain vascular system of different arteries and veins in 3D. Red indicates arteries, and blue indicates veins. The vascular names are all marked in black. The names of brain regions are marked in gray. **(A)** The distributions of Acer and its major branches are mainly located between the CP. **(B)** The distributions of Mcer and its major branches, which mainly extend into the CP and the outside of the pial surface. **(C)** The distributions of Pcer and its major branches, which mainly penetrate the HIP and TH below the HIP. **(D)** The distributions of Pcer and Vert and their major branches. The annotated branches of Vert mainly extend to both the surface and interior of the CB, P, and MY. **(E)** The distributions of Ach and Achv and their major branches, which are located in the rostral area of the HIP. **(F)** The distributions of Lhiv and its branches, which penetrate into the upper portion of the HIP and TH below the HIP. **(G)** The distributions of the thalamostriate vein (Thsv) and Gcv inside the brain, which extend to the caudal part of the CP and the rostral part of the TH along the middle area of the HIP. **(H)** The distributions of Mcolv and Lcolv and their branches. Mcolv is mainly located in the rostral aspect of the CB and the lateral aspect of the MB below the HIP, whereas Lcolv penetrates the lower portion of the HIP. **(I)** The distributions of the pial surface veins and the connected cortical penetrating veins. The penetrating veins presented here extend to the subcortical area. The list of abbreviations and full names of the brain regions is presented in Supplementary Table 1, and the tree of abbreviations and full names of the vessels is presented in Figure 4. D, Dorsal; V, ventral; L, left; R, right; A, anterior; P, posterior.

**Figure 4:**
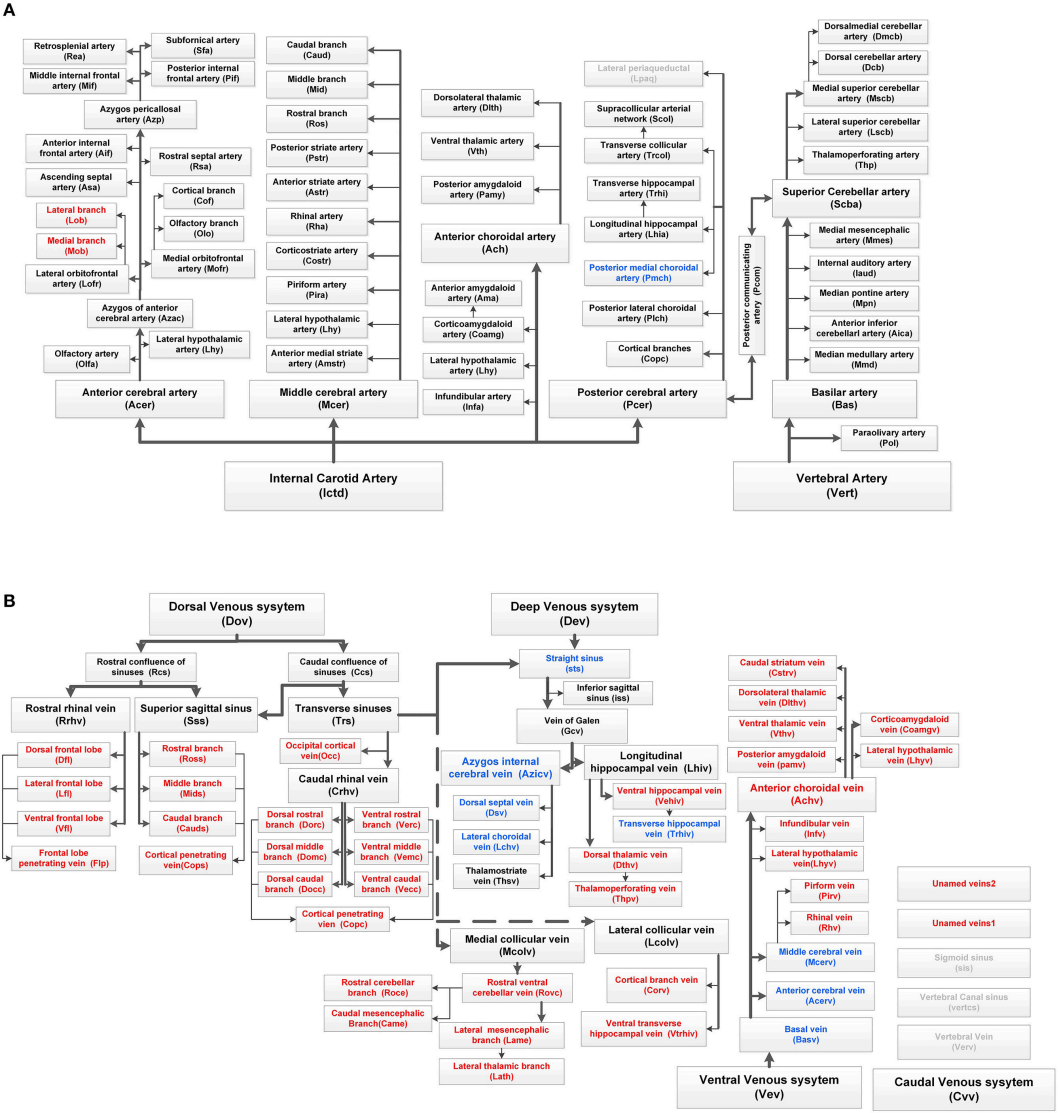
Complete tree-like structures and annotations of the arterial and venous vascular system in the whole mouse brain. To ensure readability, the tree-like structure is arranged in the sagittal direction, in which left represents rostral, right represents caudal, up represents dorsal, and down represents ventral. The full names of vessels are presented in each box, and abbreviations are presented in brackets. **(A)** Tree-like structure of the arterial system. **(B)** Tree-like structure of the venous system. Vessels in black indicate the reported branches of the mouse brain vascular system study. Vessels in blue indicate the branches reported in the rat brain vascular system study but not reported in the mouse brain. Vessels in red indicate the branches not reported in the rat or mouse brain. Vessels in gray indicate the missing branches in our data.

We annotated the traced cerebral vascular system of arteries and veins based mainly on the following naming rule (Supplementary Figure 5): “orientation-brain region-artery/vein.” The fine 3D distribution and annotation results of the traced cerebral vascular system in an individual mouse brain were presented (Figures 3, 4). The traced cerebral vascular system included, six main arteries: Acer, Mcer, Pcer, and anterior choroidal artery (Ach) originated from the internal carotid artery (Ictd) and the basilar artery (Bas), superior cerebellar artery (Scba) originated from the vertebral artery (Vert). Twelve main veins in four areas: Rrhv, Sss, Trs, and Crhv in dorsal area, the basal vein [Basv: anterior cerebral vein (Acerv), middle cerebral vein (Mcerv)], anterior choroidal vein (Achv) in the ventral area, the vein of Galen (Gcv), azygos internal cerebral vein (Azicv), longitudinal hippocampal vein (Lhiv), medial collicular vein (Mcolv), and lateral collicular vein (Lclov) inside the brain and the Unamed vein [Unamedv: (Unamedv1, Unamedv2)] in the caudal area. To show the positions of the vessels clearly, the contours of the corresponding brain regions were shown as well.

In the arterial system, Acer is the rostral branch of the Ictd. Its main trunks head dorsally to the pial surface along the midline area, and the branches are mainly located in the HY, striatum (CP), frontal lobe (FL), olfactory bulb (OLF), and dorsal part of the cerebral cortex. Mcer is the middle branch of the Ictd. The three branches of Mcer extend on the pial surface in the ventral to dorsal direction, together with branches of Acer in the pial surface in the dorsal to ventral direction, cover almost all of the cerebral cortex. The branches of Mcer inside the brain are mainly located in the HY, CP, and amygdala (AMY). Ach originates from the Ictd and is located near Mcer in the caudal direction. The branches of Ach head in the ventral to dorsal direction to the dorsum of the TH through the AMY, HY, TH, and choroid. Pcer is the caudal branch of the Ictd. The main trunk of Pcer heads dorsally through the area between the cerebral cortex and midbrain (MB), and four large branches extend to the medial choroid, lateral choroid, ventral HIP, and SC, respectively, while pial surface branches in the caudal to rostral direction supply the cerebral cortex not covered by Acer and Mcer. Bas originates from the fusion of the two Verts, and is located in the ventrum of the medulla (MY) and pons (P). There are also branches that penetrate the MY and P in the ventral to dorsal direction. Then, Bas extends to the ventral part of the TH to form Scba, which extends in the ventral to dorsal direction into the rostral area of the cerebellum (CB). The Scba supplies blood to the CB and is connected with the Pcer with the posterior communicating artery (Pcom), forming a cooperation between the two blood supply sources.

In addition, in the venous system, in the dorsal area, the branches of Rrhv head in the dorsal to ventral direction to supply the FL and OLF. The main trunks of the Sss travel along the midline in the pial surface, and the branches cover the dorsal cerebral cortex and subcortex. Trs is located in the caudal area of the cerebral cortex, and heads ventrally to form Crhv, which extends in the caudal to rostral direction through the lateral midline on the pial surface, together with Rrhv, Sss, and Trs and covers almost all the pial surface. The penetrating branches of Crhv extend to the CP and HIP. In the ventral area, Basv branches off Acerv and Mcerv, which are almost parallel to Acer and Mcer, respectively, but the extending distance to the dorsal area are short. Penetrating branches of Basv extend to the cerebral cortex, CP, HY, AMY, TH, HIP, and MB from the ventral area. Achv, which is connected to Basv, extends in the ventral to dorsal direction. Achv runs almost parallel to Ach. Inside the brain, Gcv produces Azicv and Lhiv. Azicv heads in the opposite direction of Acer to the corpus callosum (cc), CP, TH, and HIP, along the midline area. The main trunks of Lhiv are located between HIP and TH, and branches also penetrate HIP and TH. Lcolv covers the lower portion of HIP. The main trunks of Mcolv exist between MB and cortex, and branches will supply CB, MB, and TH. In caudal area, Unamedv contains Unamedv1, located in dorsal CB, and Unamedv2, located in dorsal P, and penetrates into cerebellum and pons.

Detailed spatial distributions and extending patterns of all annotated vessels are described in the Supplementary Notes. We first provide 3D distributions and annotation results for the fine vascular system of both the arteries and veins in the whole mouse brain. The annotations of arteries and the large veins are the same as those reported in the literature (Cook, 1965; Dorr et al., 2007; Lin et al., 2009; Scremin, 2012), but we find and annotate some new branches, which are presented in Figure 4, especially in veins.

Combined with the brain regions provided by the cytoarchitecture, we accurately located each traced artery and vein. The extending directions, detailed extending paths, numbers, covered areas, and the relationships with brain regions of the vascular branches could be determined. Furthermore, we could acquire quantitative morphological parameters based on the vectorized results, such as the length, diameter, order, volume, density, and bifurcation number of the vascular branches.

### Creating the cerebral vascular system atlas

**Figure 5:**
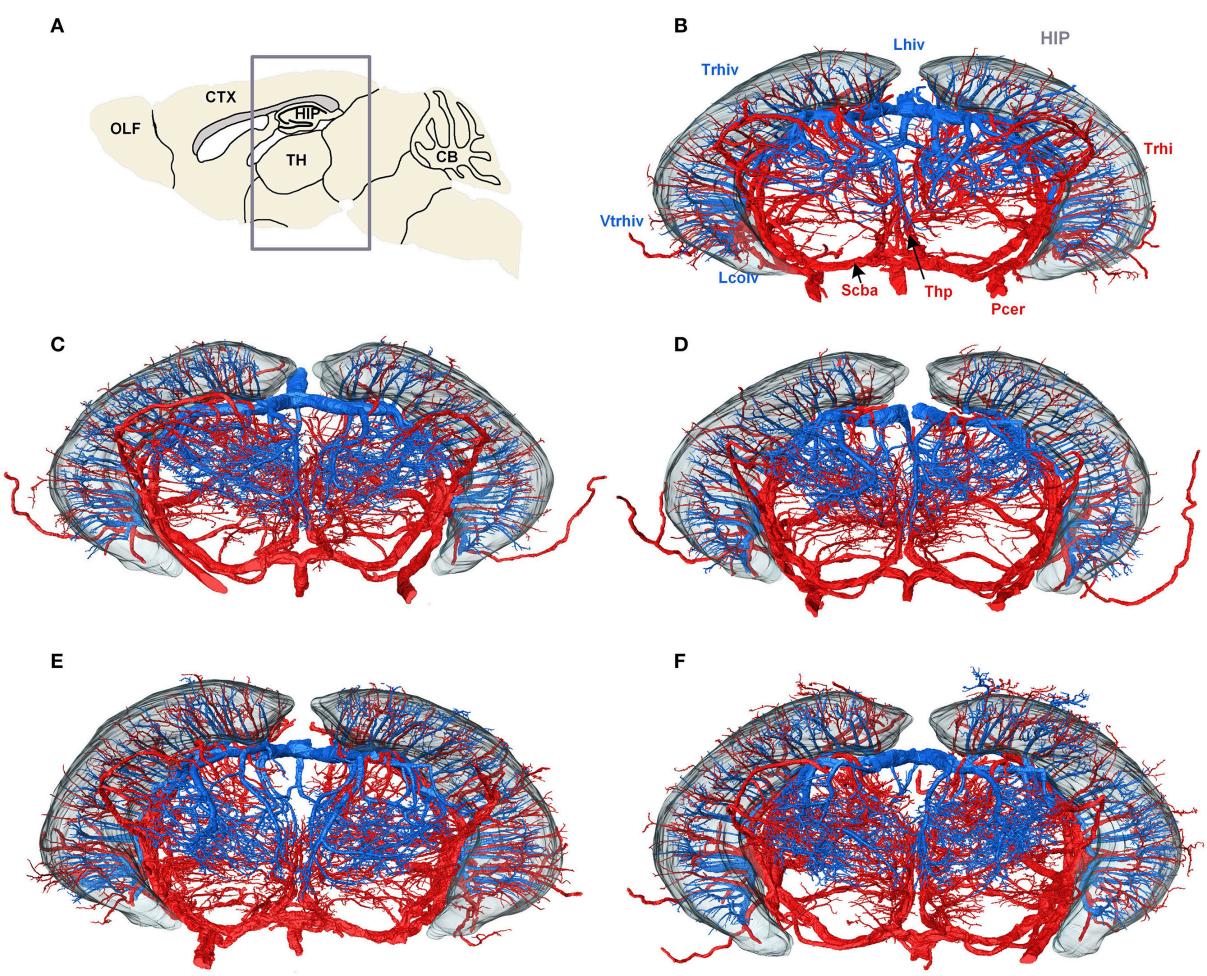
Vascular conserved distribution results across five mouse brains. **(A)** Sagittal slice used for localization; the gray box shows the range in the rostral to the caudal direction of the 3D datasets in **(B–F)**. **(B–F)** Three-dimensional reconstructed results for Lhiv, Lcolv, and Pcer in five mouse brains. To identify the position and direction of the vessels in 3D, the contour of the HIP is shown as well. In **(B)**, the main branches of Pcer and Scba are marked in red, while the branches of Lcolv and Lhiv are marked in blue, and brain regions are marked in gray. A list of abbreviations and full names of the brain regions is presented in Supplementary Table 1, while the tree of abbreviations and the full names of vessels is presented in Figure 4.

To create a conserved cerebral vascular system atlas, we reconstructed and annotated the cerebral vascular system in five C57BL/6 mouse brains. The reconstructed structures of Pcer, Scba, Lhiv, and Lcolv in these brains were presented to illustrate the conserved vascular distributions among different individuals (Figure 5). Trhi always originates from Pcer and penetrates the HIP in the caudal to rostral direction, and the thalamoperforating artery (Thp) always originates from Scba and emanates in the ventral to dorsal direction in the TH below the HIP. Trhiv originates from Lhiv, and Vtrhiv originates from Lcolv in the upper and lower portions of the HIP, respectively. The thalamoperforating vein (Thpv) emerges from Lhiv and penetrates into the TH from the dorsal and lateral areas to form an anastomotic distribution with Thp. The qualitative results indicate that the distributions of arteries and veins are conserved among the different mouse brains.

**Figure 6:**
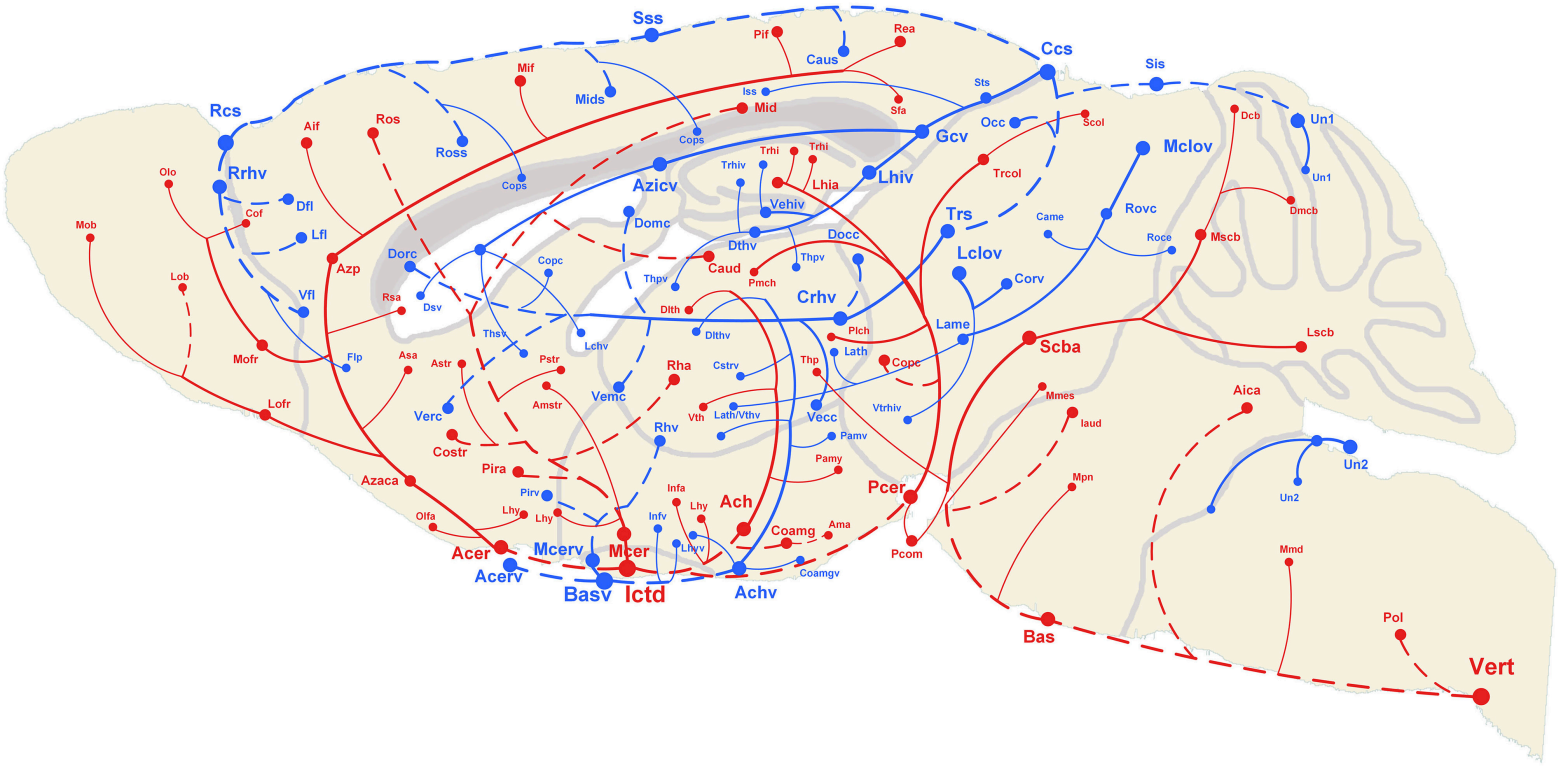
Distribution map of the arterial and venous vascular system in the whole C57 mouse brain. Abbreviations and trending curves of the arteries and veins are presented in red and blue, respectively. Both the arteries and veins are presented in 3 levels using 3 different sizes from large to small of the font, sphere and link curves, which respectively represent the size of the vessel diameter, original/terminal points, and extending directions. The dotted curves and full curves respectively represent the vascular branches in the pial surface and inside the brain. A tree of abbreviations and full names of the vessels is presented in Figure 4.

**Figure 7:**
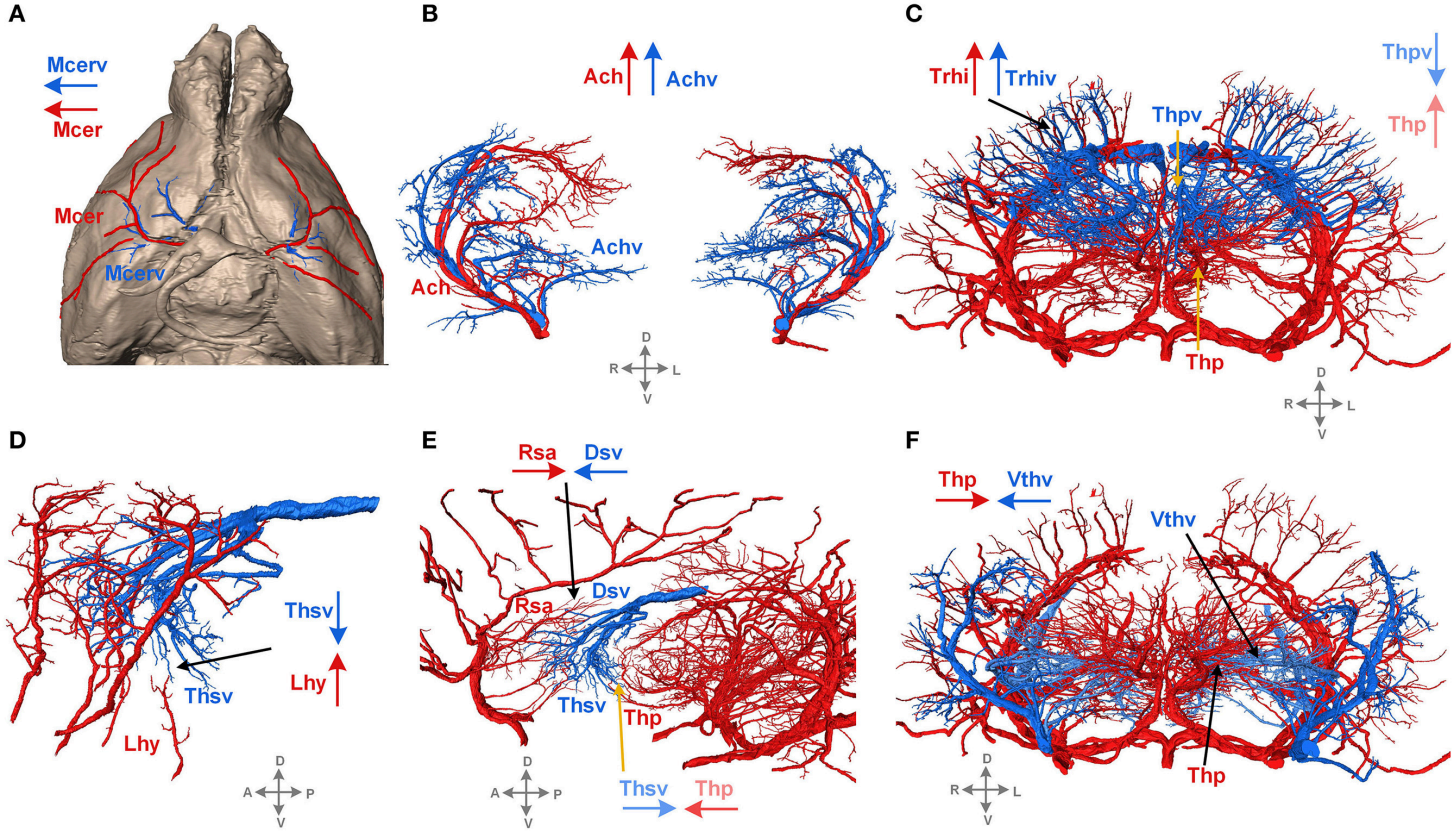
Typical distribution patterns of arteries and veins. Arteries are presented in red, and veins are in blue. The arrows in red and blue indicate the characteristics of the extending direction. D, dorsal; V, ventral; L, left; R, right; A, anterior; P, posterior. A tree of abbreviations and the full names of the vessels is presented in Figure 4. **(A)** The distribution patterns of vessels on the brain surface. **(B–F)** The distribution patterns of vessels inside the brain.

The 3D reconstructed cerebral vascular system in the brains mentioned above were combined to obtain conserved distribution patterns of the brain-wide arterial and venous vascular system in the form of a 2D brain map (Figure 6). We classified the arteries and veins according to the bifurcation position, starting from the origin of large vessels and the diameter of the branches from large to small. To ensure the readability of the image, Figure 6 show the distributions and extents of the arteries and veins at 3 levels (about D > 90 μm, 40 < D < 90 μm, D < 40 μm) with diameters from large to small. The results show the distribution patterns of both the arteries and veins (Figure 6) and the relationships between the arteries and veins (Figure 7). The cortical penetrating arteries and penetrating veins, Ach and Achv, and Mcer and Mcerv, are parallel in the cortical area. By contrast, in the subcortical area, Thp and Thpv and Thsv and the lateral hypothalamic artery (Lhy) anastomose in the dorsal to ventral direction; rostral septal artery (Rsa) and dorsal septal vein (Dsv) anastomose in the rostral to caudal direction; Thp and the ventral thalamic vein (Vthv) anastomose in the medial to lateral direction, and Trhi and Thriv, and Trhi and Vtrhiv distribute alternately. However, there is no relationship of the distribution patterns between most of the arteries and veins, indicating that it is necessary to study both arteries and veins. Based on our results, the locations, extents, connecting patterns, and coverage area of each artery and vein in the whole mouse brain can be observed directly. We first provide a fine mouse cerebral vascular system atlas in stereotaxic coordinates of both the arteries and veins at three levels, which will benefit the understanding of the mouse cerebral vascular system, especially to redefine the knowledge of the venous vascular system.

### Distributing patterns between the vasculature and brain regions

The brain regions of different function are correlated with neural activities. When a sensory is inputted to a brain region, the flow of blood will increase in the brain function imaging (Grinvald et al., 1986; Blinder et al., 2013). But the knowledge of real vascular distribution patterns and the vascular density within different brain regions are rather sparse (Hirsch et al., 2012). The areas that are affected when an artery or vein is infarcted, that is the distributing patterns between vessels and brain regions, provide essential information for the study of clinical and cerebral vascular diseases. Previous studies regarded the brain regions as the marks to orientate the vessels and there have been no complete distribution proportion results among blood vessels and brain regions of the whole mouse brain.

**Figure 8:**
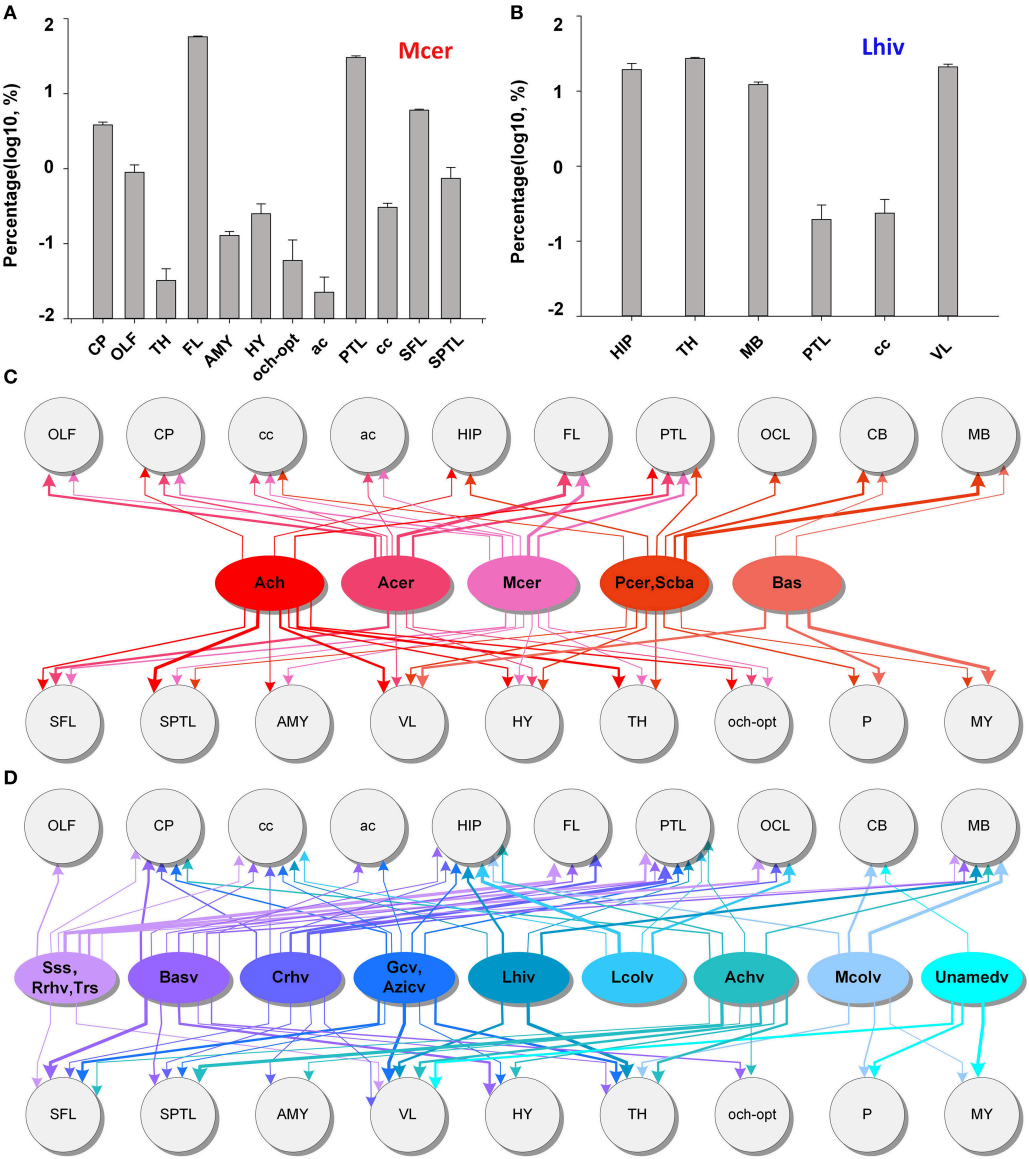
Distributing patterns between the arterial and venous vascular system and brain regions in the whole mouse brain. **(A,B)** The statistical results for the volume distributions of Mcer and Lhiv within different brain regions. The segmented brain regions are presented on the horizontal axis, and the distribution proportions (after log10 transformation) of vessels in the corresponding brain region are presented on the vertical axis. **(C,D)** Distributing patterns of arteries and veins with the main brain regions. The lines and arrows between the vessels and brain regions indicate the branches that extend to the brain region, while the line thickness represents the values of distribution proportions. A list of abbreviations and the full names of the brain regions is presented in Supplementary Table 1, while a tree of abbreviations and the full names of vessels is presented in Figure 4.

To acquire the distributing patterns of the arteries and veins with brain regions, we obtained quantitative statistics of the mean volume distribution proportions (Supplementary Figure 7) of each artery and vein within different brain regions (see the section Materials and Methods) (*n* = 5). The quantitative statistical results for Mcer and Lhiv (Figures 8A,B) presented as samples of arteries and veins, respectively. The statistical results show that Mcer is mainly distributed in the FL, parietal temporal lobe (PTL) and CP, in which the cortex represents 80.6% in all and the CP represents the maximum portion in the subcortex of 3.8%. Lhiv is mainly distributed in the HIP, TH, MB, and cc; these 4 regions account for ~80% of Lhiv. Based on the distributions of different arteries and veins in different brain regions, we constructed the distributing patterns between the vessels and brain regions (Figures 8C,D). Only the distributing patterns results of Ach and Achv with the brain regions are consistent (11 brain regions with one difference), while the results for the other arteries and veins are not. Each artery or vein connects to one or more brain regions, and there are weakly connected brain regions. The distributing patterns of individual vessels different from each other. Each brain region is connected to many different arteries and veins to ensure the blood supply, indicating that the fine and accurately located vascular system of both the arteries and veins and the distributing patterns between cerebral vessels and brain regions in whole mouse brain are of great importance to the study of vascular distributions and brain function. Here we provide the relation between the vessels and brain regions, and we will provide the vascular density in different brain regions in the next part.

### Quantification of the vascular system (arteries and veins) in the whole brain

To quantitatively study the conserved distributions of both arteries and veins among different individuals, we counted the branch numbers of 6 large arteries and large veins in 4 areas of 5 mouse brains. The statistical results show that the standard deviation values are small for both arterial and venous branch numbers at the first and second levels with diameter D > 90 μm and 40 < D < 90 μm, respectively (Table 1). The mean values are similar between the left and right brain of both arteries and veins at the first and second levels, indicating that the branches of arteries and veins at the first and second levels are conserved both among different mouse brains and between the left and right brain. No correlation is observed between the statistical results for the arteries and veins. The branch numbers increase with the level increases in both arteries and veins. The maximum branch number is observed in both arteries and veins at the third level with diameter D < 40 μm. The differences in the mean and standard deviation values increase at the third level, reflecting differences in vascular distribution among the different mouse brains and between the left and right brains. These findings confirm traditional beliefs about mouse brain vessels that the large vessels are more conserved than the small vessels (Semenza, 2007; Geudens and Gerhardt, 2011). Detailed statistical results are listed in Table 1.

**Table 1:** Statistical table of the branch numbers of arteries and veins at 3 levels.

**Arteries**	**L**	**R**	**Veins**	**L**	**R**
**Acer**			**Dov**		
1st	1	1	1st	4	4
2nd	7.6 ± 0.5	7.4 ± 0.5	2nd	17.0 ± 2.9	16.2 ± 1.6
3rd	10.3 ± 0.9	9.5 ± 0.8	3rd	23.2 ± 4.0	21.6 ± 3.6
**Mcer**			**Dev**		
1st	1	1	1st	4.5	4.5
2nd	6.6 ± 0.5	6.2 ± 0.8	2nd	4	4
3rd	12.0 ± 1.0	11.8 ± 1.6	3rd	57.3 ± 1.9	58.5 ± 2.0
**Ach**			**Vev**		
1st	1	1	1st	2.2 ± 0.4	2.2 ± 0.4
2nd	0.6 ± 0.5	0.2 ± 0.4	2nd	4.4 ± 0.5	4
3rd	4.4 ± 1.5	3.2 ± 1.3	3rd	16.0 ± 2.1	16.4 ± 2.3
**Pcer**			**Cvv**		
1st	1	1	1st	3	3
2nd	7.4 ± 0.5	8.2 ± 0.4	2nd	3	3
3rd	20.8 ± 2.2	23.4 ± 0.9			
**Scba**					
1^st^	1	1			
2nd	2.8 ± 0.4	2.8 ± 0.4			
3rd	18.7 ± 2.2	20.1 ± 1.1			
**Bas**					
1st	1	1			
2nd	2.4 ± 0.5	2.4 ± 0.5			
3rd	9.6 ± 3.5	10.2 ± 2.7			

*According to the vessel classification in Figure 4, we counted the number of vascular branches at 3 levels. L and R represent the left and right halve brain. The values in the table are the mean values and standard deviation in the arteries and veins of 5 mouse brains at 3 levels*.

### Quantification of the microvessels in brain regions

Functional interaction between the cerebral vessels and brain tissues occurs in the microvessels, where the neurovascular unit is generated (Zhang et al., 2012) that maintains the energy metabolism of neurons and glial cells. To facilitate functional study of the cerebral vessels, we further analyzed the microvessels (Figure 8C, Supplementary Figures 8a3–e3). The capillaries were separated with the diameter size of 8 μm (Michaloudi et al., 2006).

**Figure 9:**
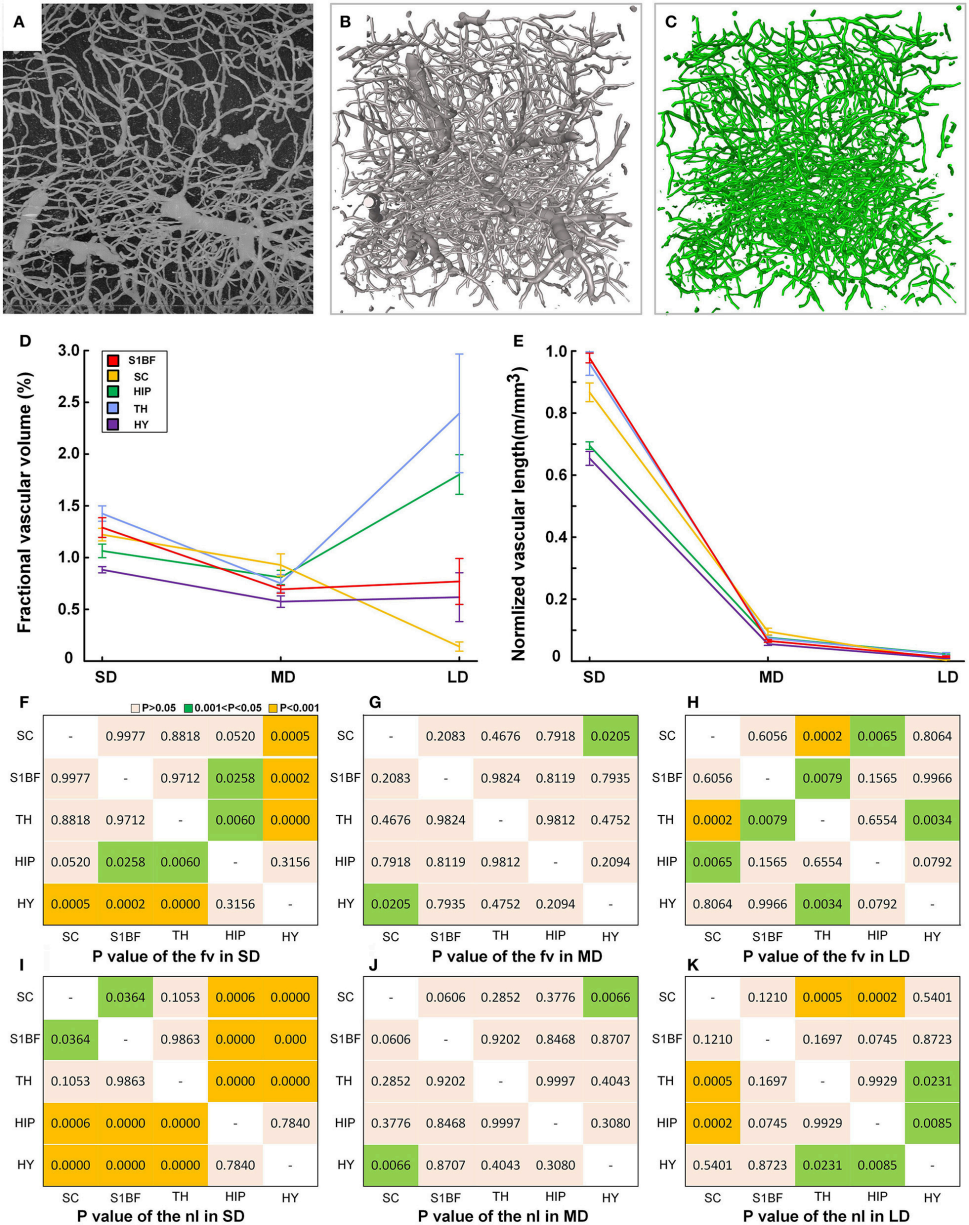
Quantitative statistical results for the capillary density in the cortical and subcortical areas of the mouse brain. **(A)** Maximum projection of 200 μm to show the vessels in HIP (image size of 400 × 400 μm). **(B)** The reconstructed fine vascular network in HIP (stack size of 400 × 400 × 400 μm). **(C)** Separated capillaries in HIP with diameter size of 8 μm (stack size of 400 × 400 × 400 μm). **(D,E)** Quantitative statistical results for the fractional vascular volume (Fv) and normalized vascular length (Nl) in the S1BF, SC, HIP, TH, and HY. SD, small-sized vessel (D < 8 μm) density; MD, medium-sized vessel (8 < D < 20 μm); LD, large-sized vessel (D > 20 μm) density. **(F–H)** The detailed diversity distributions (*P*-values) among 5 brain regions of fv in SD, MD, and LD. **(I–K)** The detailed diversity distributions among 5 brain regions of nl in SD, MD, and LD; S1BF, primary somatosensory cortex, barrel field; SC, superior colliculus; HIP, hippocampus; TH, thalamus; HY, hypothalamus.

We provided fine microvascular structures (Figures 9A–C) and statistical vascular densities in five typical brain regions, as calculations at the microvessels are very time consuming. Based on the cytoarchitecture (Supplementary Figures 8a–e), we selected the S1BF, SC, TH, HIP, and HY as five brain regions in the cortical and subcortical areas as study subjects in three mouse brains. One image stack with a size of 400 × 400 × 400 μm (about 1.4 GB) was chosen in both the left and right brains of three mice (*n* = 6). Fractional vascular volume (Fv) and normalized vascular length (Nl) were calculated in small-sized vessels (capillaries) (SD, D < 8 μm), medium-sized vessels (MD, 8 < D < 20 μm), and large-sized vessels (LD, D > 20 μm; Michaloudi et al., 2006), and the difference in distribution of the vascular network among five brain regions was analyzed by One-way ANOVA. The results (Figures 9D,E) show that the Fv and Nl of the capillaries are both high in the TH because all information transmission to the cortex passes through the TH (Sherman and Guillery, 2001). The density values of medium-sized and large-sized vessels are lowest in the HY, which contains mainly small-sized and medium-sized vessels. The statistical results reveal that the differences of vascular density mainly exist mainly in small-sized vessels and large-sized vessels among different brain regions (Figures 9F–K), especially in small-sized vessels (capillary). And the connected paths between the arterioles and venules, have the potential to be used for calculating blood flow in the brain.

## Discussion

The scientific advances of our work reflects in three points: (1) We reconstructed, annotated and analyzed the vascular system atlas of both arteries and veins in the whole brain, using the data with voxel size of 5 × 5 × 5 μm^3^. And we unveiled some new branches, especially in veins, which have redefined the knowledge of the venous vascular system; (2) The structure of the mouse brain vasculature is conserved across individuals in larger vessels with diameter large than 40 μm, the differences begin to emerge in small vessels with diameter < 40 μm; (3) We reconstructed and demonstrated the differences of microvessels through calculating the vascular density, using data with voxel size of 0.35 × 0.35 × 1 μm^3^, not only in the cerebral cortex, but also in the subcortex.

We have acquired high-resolution datasets of vascular structures and cytoarchitecture at a voxel resolution of 1 μm for the whole mouse brains by taking the advantages of MOST combined with a modified Nissl staining method. MOST system performs simultaneous thin sectioning and imaging while recording the image coordinates for automatic aligning. The Nissl staining is conventionally used to get cytoarchitecture in brain tissues (Dong, 2011; Paxinos and Franklin, 2012; Windhorst and Johansson, 2012). We modified the traditional Nissl staining method to label both cytoarchitecture and vascular structures simultaneously in one and the same whole mouse brain (Wu et al., 2014). The cells and vascular walls were stained in dark, while the vascular lumen were not stained and presented in white (Supplementary Figure 1). The datasets of India-ink staining and vascular casting (Heinzer et al., 2006, 2008; Ryang et al., 2011; Xue et al., 2014; Pesavento et al., 2017) have high image quality and contrast but only the vascular structure can be acquired, while the Nissl stained dataset can provide the cytoarchitecture at the same time. The cytoarchitecture was used to orientate the vessels and get the brain regions. Here, we accurately located each arteries and veins in 3D of the whole mouse brain based on the Nissl stained data, and coordinates generated by the cytoarchitecture can be used to reliably find specific arteries or veins (Supplementary Figure 9). Meanwhile we acquired the distributing patterns between the vasculature and brain regions. But only the arteries and veins were reconstructed and analysis in the whole brain scale, and the microvessels were only reconstructed and analysis in five brain regions.

We first constructed the precise mouse cerebral arterial and venous vascular system atlas in stereotaxic coordinates. The minimum diameter of the reported arterial system is ~20 μm (Ghanavati et al., 2014), while the terminal branches of arteries and veins acquired here were all as small as 8–10 μm. Comparisons of our constructed treelike structures of the mouse brain arterial and venous vascular system with the reported vascular system of mouse and rat brains are shown in Figure 4, and the results indicate that we have constructed the fine venous vascular system and described many new structures of veins. We have redefined the knowledge of the venous vascular system from the traditional primary large veins to the fine venous structure at three levels; together with the arteries, we have constructed the precise mouse cerebral vascular system. There are some discrepancies between our results and those of previous studies of arterial branches in mouse and rat brain vascular systems (Cook, 1965; Dorr et al., 2007; Scremin, 2012). For example, Pcom was reported to be absent or poorly formed in the C57BL/6 mouse brain (Barone et al., 1993; Dorr et al., 2007), but in our C57BL/6 mouse brain, the Pcom was found in both sides of 3 brains and in one side of 2 brains. In addition, previous studies suggested that Pcom was connected between the Pcer and Ictd (Scremin, 1995; Dorr et al., 2007), but in our data, Pcom was connected to Pcer and Scba. Pcer and Thp originate from Scba and Pcer, respectively, in the CBA mouse brain (Dorr et al., 2007), but from Ictd and Scba in our C57BL/6 mouse brain. The results here will benefit the understanding of the arterial and venous vascular system in whole brains of different strains of mice.

The perfusion patterns of vessels in brain regions are important foundations for clinical application (van Laar et al., 2008). The results for the distributing patterns between the cerebral vascular system and brain regions reveal the relationships of the supplying arteries and draining veins with brain regions. Based on these results, we can determine the areas that are mainly supplied by each artery and the areas from which each vein mainly drains blood. The quantitative distributing patterns between the vascular system and brain region in the whole mouse brain have not been reported previously; brain regions were only considered to locate the cerebral vascular system (Scremin, 1995, 2012; Dorr et al., 2007). The quantitative relationships between the cerebral vascular system and brain regions will provide references for identifying occlusion sources both experimentally and clinically.

Energy metabolism between the bloodstream and brain tissues mainly occurs in the capillaries (Zhang et al., 2012). Traditionally, statistical results for vascular density based on high resolution datasets have mainly been acquired in cortical areas (Tsai et al., 2009; Blinder et al., 2013) and vascular densities were used to study the relationships between vessels and cells (Tsai et al., 2009; Wu et al., 2014). Here, we presented the statistical microvasculature densities not only in the cerebral cortex, but also in the subcortex, based on datasets with a voxel resolution of 1 μm and studied the vascular distribution differences within five brain regions. The calculated results for the vascular density were revised according to linear shrinkage (Wu et al., 2014) of 25.9 ± 1.6% to correct for the isotropic shrinkage caused by the sample preparation. From the statistical results we can get that the capillary densities of TH and S1BF are high, because all information transmission to the cortex passes through the TH (Sherman and Guillery, 2001) and the S1BF are correlated with the whiskers (Diamond et al., 2008), which are used to detect the environment in the night. But the capillary density of HIP is low, while the HIP plays important roles in the consolidation of information from short-term memory to long-term memory, and in spatial memory that enables navigation.

High resolution datasets, including the capillary bed, were acquired with MOST. The complex real connected path of capillaries has the potential to be used for calculating blood flow in the brain (Reichold et al., 2009; Linninger et al., 2013) to provide insights on metabolism and neuro-vascular coupling (Iadecola and Nedergaard, 2007). Here, we only provide the reconstructed and statistical results of the fine microvessels within brain regions, limited by the computing power. With the development of the analysis algorithms and hardware for the large data, we will continue our detailed reconstruction and comparative study about microvessels across both genders, different strains and different developmental phases in the future work.

The reconstruction and calculation of the arteries, veins and capillaries of the brain have demonstrated the capacity of our platform of acquiring and analyzing of the vessels at different levels. Our comprehensive platform provides important basic standard resources for the study of brain function and brain diseases. With further development of sample preparation and imaging techniques, the methods and processes could be used for the reconstruction of the vascular system in the brains of other mammals, including the human brain.

## Author contributions

HG and QL: conceived and designed the project and coordinated activities from all authors. ZY, BL, and XL: designed the modified Nissl staining method; AL: designed the imaging system MOST, and TJ used the MOST to acquired datasets; JP: performed preprocess to the data; BX and YL: performed the segmentation of the vessels and analyzed the data; TX, XL, and BL: give help with stereotaxic analysis. BX, HG, QL, SC, TX, XY, and YL: wrote and modified the manuscript.

### Conflict of interest statement

The authors declare that the research was conducted in the absence of any commercial or financial relationships that could be construed as a potential conflict of interest.
