# Supplementary material for: Precise Cerebral Vascular Atlas in Stereotaxic Coordinates of Whole Mouse Brain
Source: Front Neuroanat. 2017 Dec 19;11:128. doi: 10.3389/fnana.2017.00128 (PMC5742197; doi:10.3389/fnana.2017.00128)
Supplement: Supplementary file 1 [file Presentation1.pdf]

Supplementary Material

Precise cerebral vascular atlas in stereotaxic coordinates of whole mouse brain

Benyi Xiong[1,2], Anan Li[1,2] , Yang Lou[1,2], Shangbin Chen[1,2], Ben Long[1,2], Jie Peng[1,2], Zhongqin Yang[1,2], Tonghui Xu[1,2], Xiaoquan Yang[1,2], Xiangning Li[1,2], Tao Jiang[1,2], Qingming Luo[1,2], Hui Gong[1,2]*

*** Correspondence:** Hui Gong: huigong@mail.hust.edu.cn

1 Supplementary Figures, Tables and Notes

Supplementary Figure 1-9	1.1 Supplementary Figures
Supplementary Table 1	1.2 Supplementary Table
Supplementary Notes	1.3 Supplementary Notes

1.1 Supplementary Figures

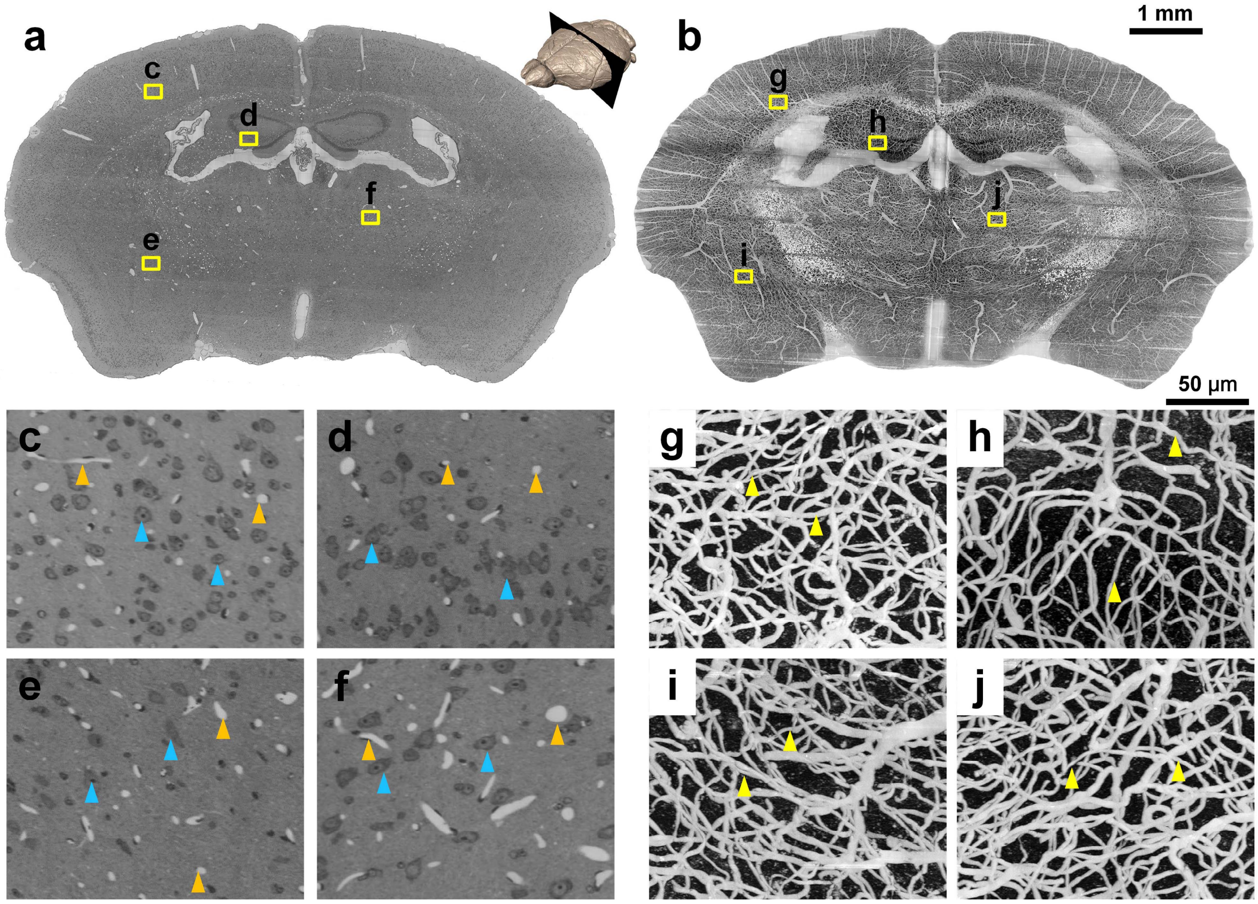

Supplementary Figure 1. Raw data of Nissl staining. (a) The preprocessed coronal section with thickness of 1 μm, and the location of the section within the brain is shown in the up right corner. (c-f) The enlarged views of the detailed information of vessels (indicated by the orange arrows and presented as white in the images) and cells (indicated by the blue arrows and presented as black in the images). (b) Maximum projection image of 200 μm (start from the images in a, posterior direction) to show the vessels. (g-j) The enlarged views of the detailed vascular information, the microvessels can be clearly visiabled (indicated by the yellow arrows).

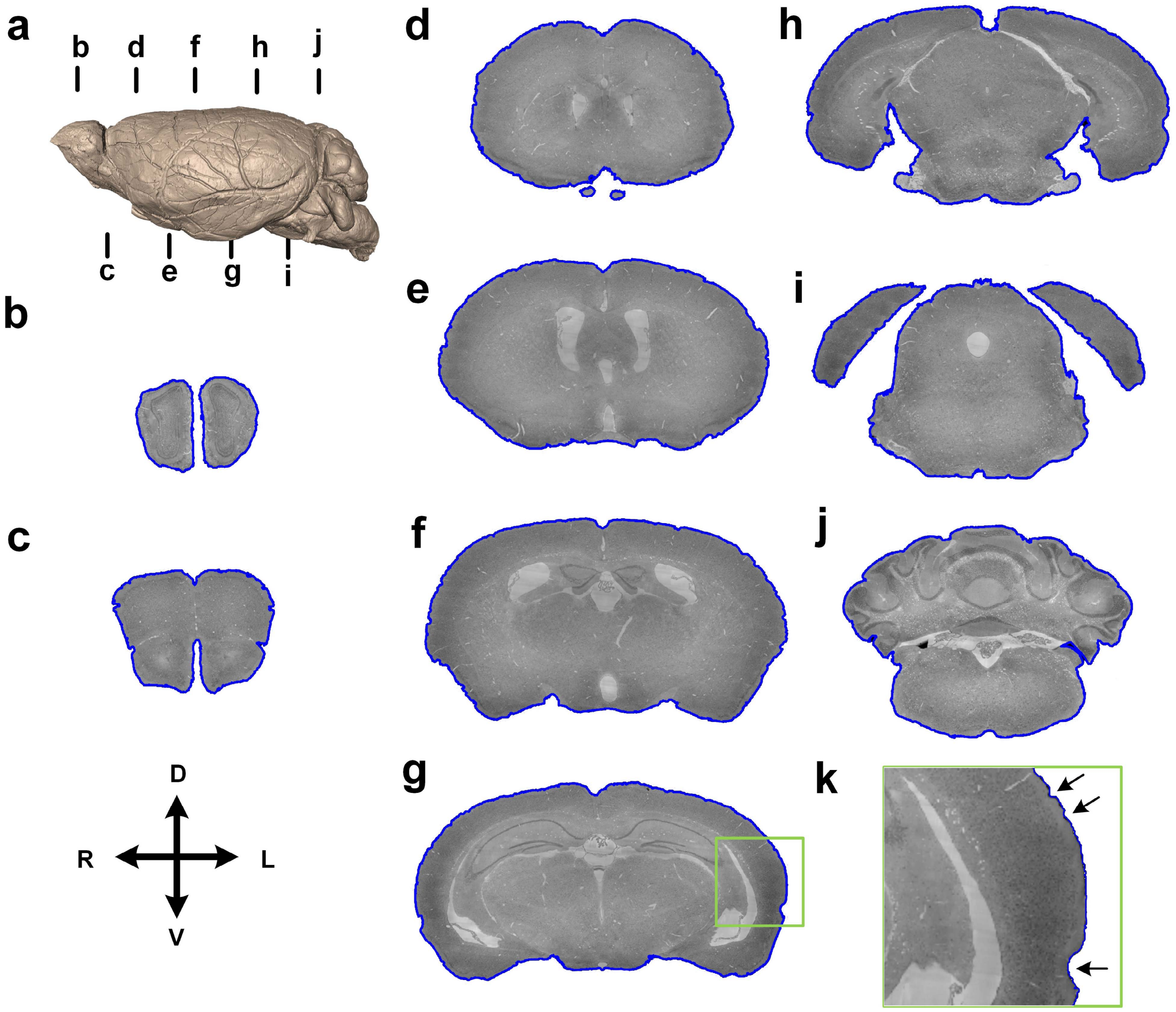

Supplementary Figure 2. Segmented brain contour in serial coronal sections. (a) Locations of the coronal sections in b-j. (b-j) Segmented 2D brain contour in serial coronal sections, presented in the rostral to caudal direction with a constant distance of 150 μm. The lines in blue are the brain contours acquired with the Otsu method and morphological operations. (k) Local enlarged views in (g); the black arrows indicate dents of pial surface vessels in 2D coronal sections. R = right, L = left, D = dorsal, V = ventral.

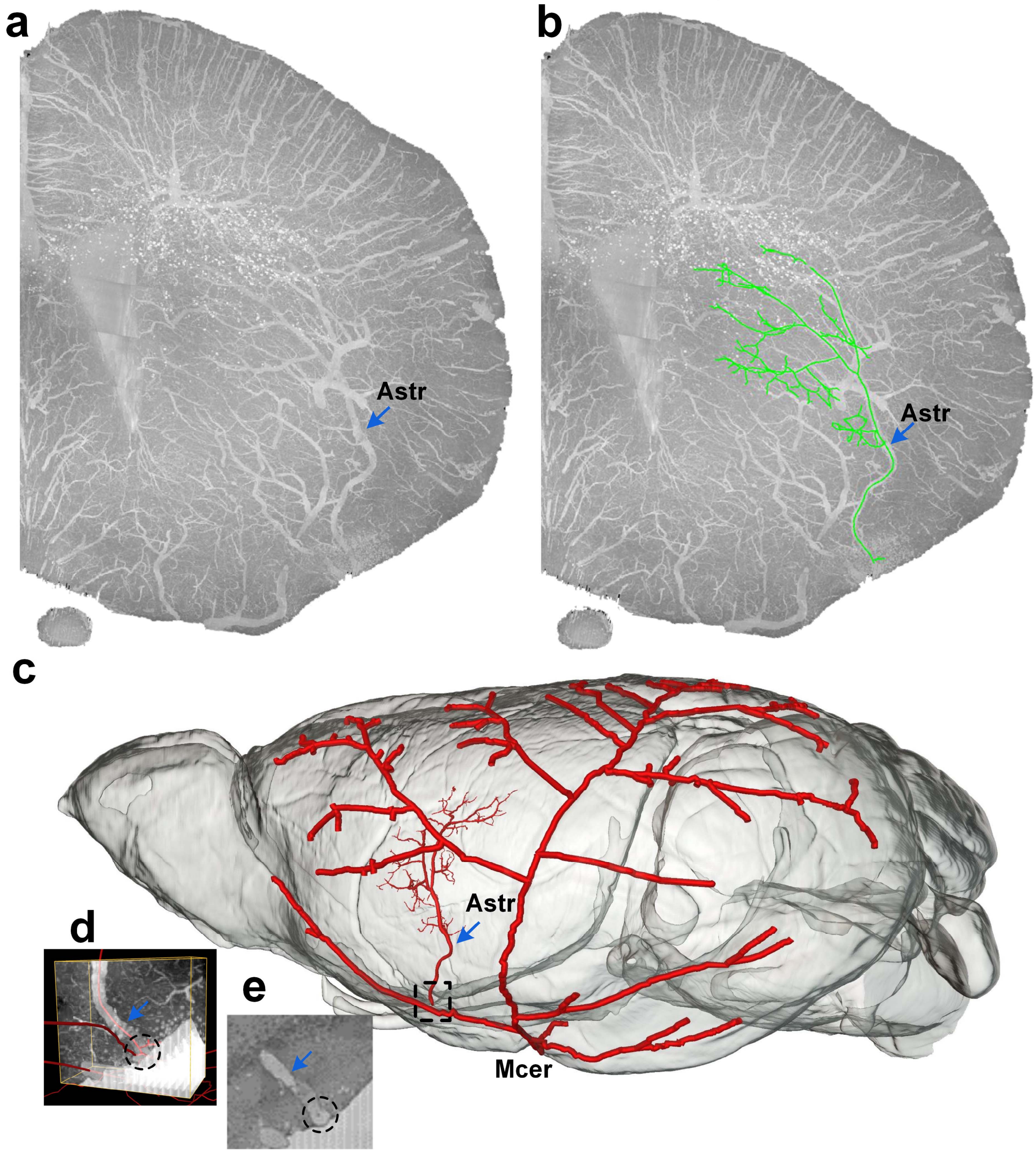

Supplementary Figure 3. Tracing of cortical penetrating vessels. The blue arrows in (a-e) indicate the cortical penetrating Astr. (a) Coronal section with maximum intensity projection (thickness of 500 μm). (b) Tracing results for Astr, which connects to the surface branch of Mcer. (c) 3D reconstructed results for Mcer and Astr. (d) Volume rendering of the original data indicated by the black box in c. (e) presents a single section of d. The black circles in (d) and (e) show the position at which Astr is connected to Mcer in the pial surface. Mcer: Middle cerebral artery, Astr: Anterior striate artery.

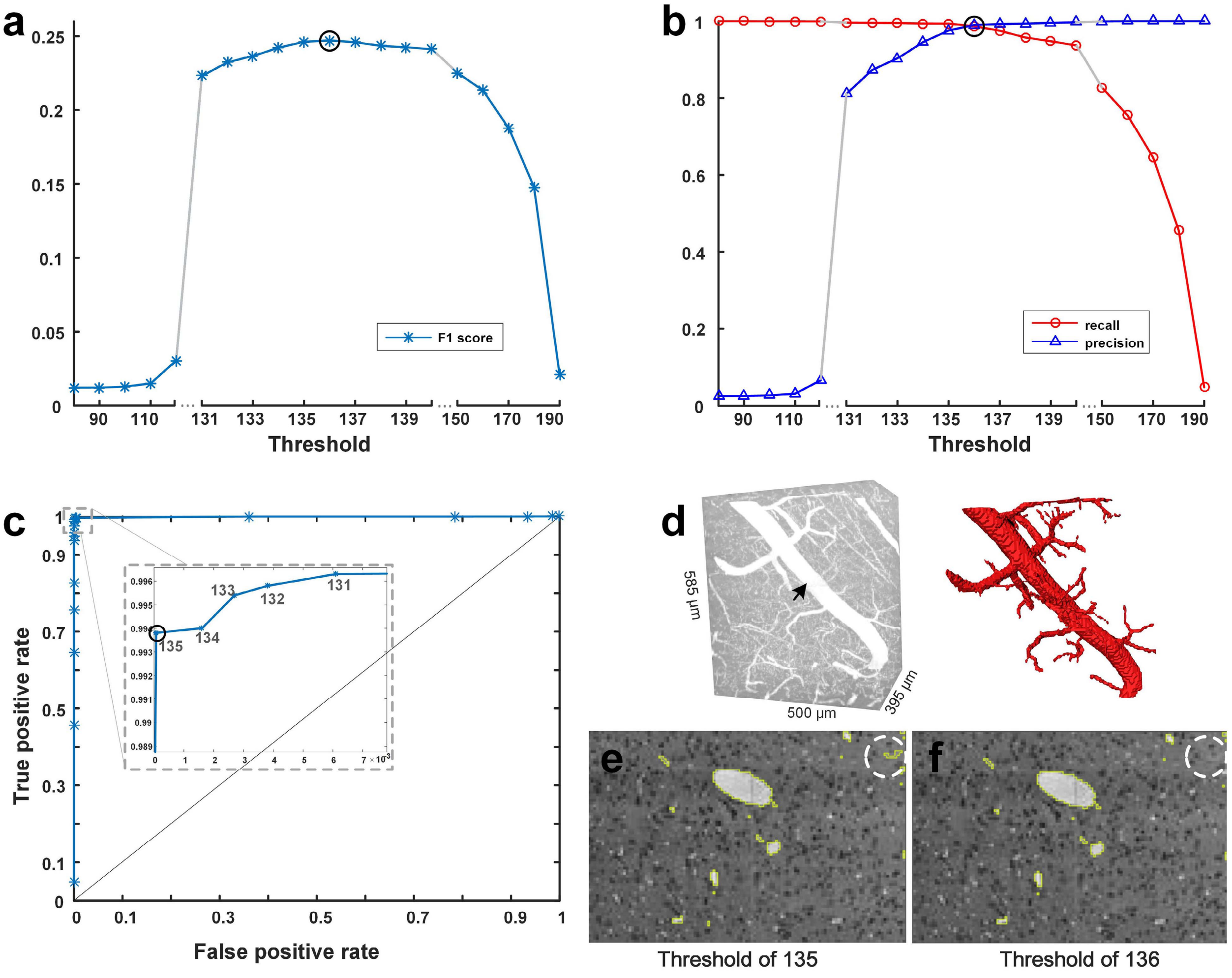

Supplementary Figure 4. Testing results of the thresholds of the dataset with voxel size of 5×5×5 μm^3. (a-c) The F1 score, Recall, Precision and ROC curves with different thresholds, and the black circles indicate the suitable threshold. (d) The 3D volume rendering results and the manual segmented ground truth of the represented data with size of 585×500×395 μm^3. (e-f) The binarized results with thresholds of 135 and 136, and the white circles indicate the incorrect segmented results.

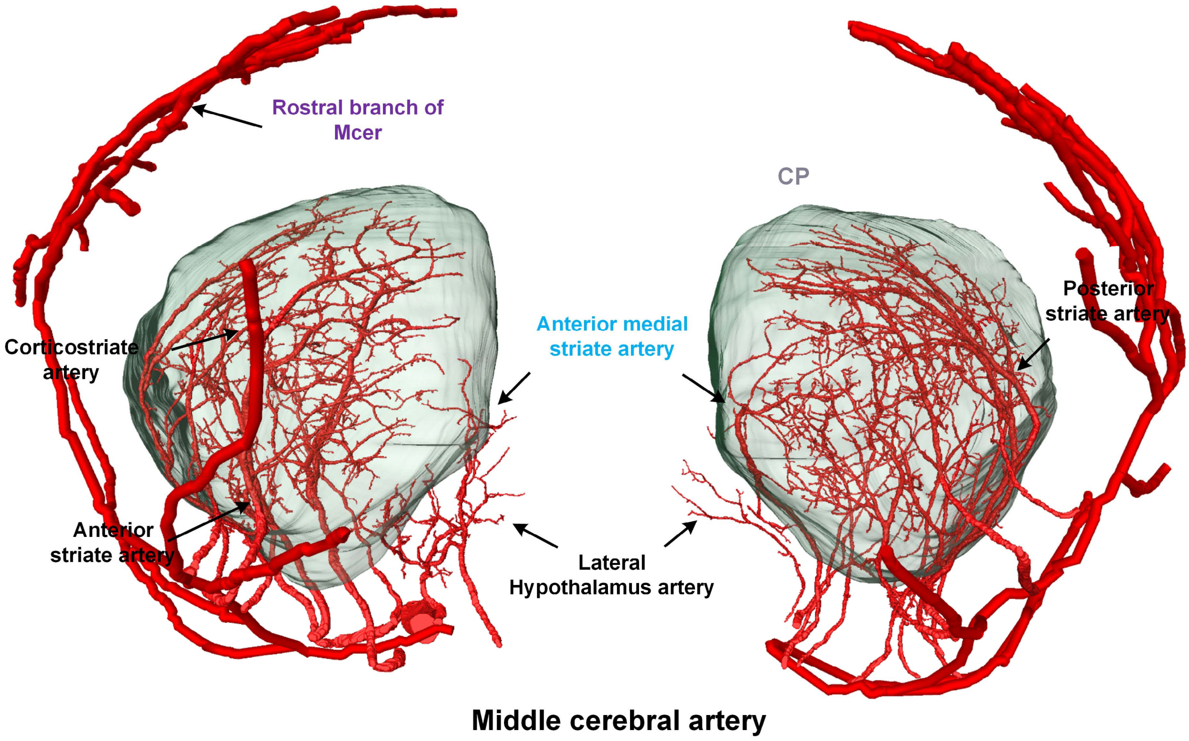

Supplementary Figure 5. Three patterns of vascular annotation. The annotation of Mcer is presented. The name of the brain region is marked in gray, while the names of vessels are in dark blue and violet. The names of vessels in black follow the rule "orientation-brain region-artery/vein", the names of vessels in blue follow the rule "orientation-orientation-brain region-artery/vein", and the names of vessels in violet follow the rule "orientation-branch of-name of connected vessel". CP = striatum, Mcer = middle cerebral artery.

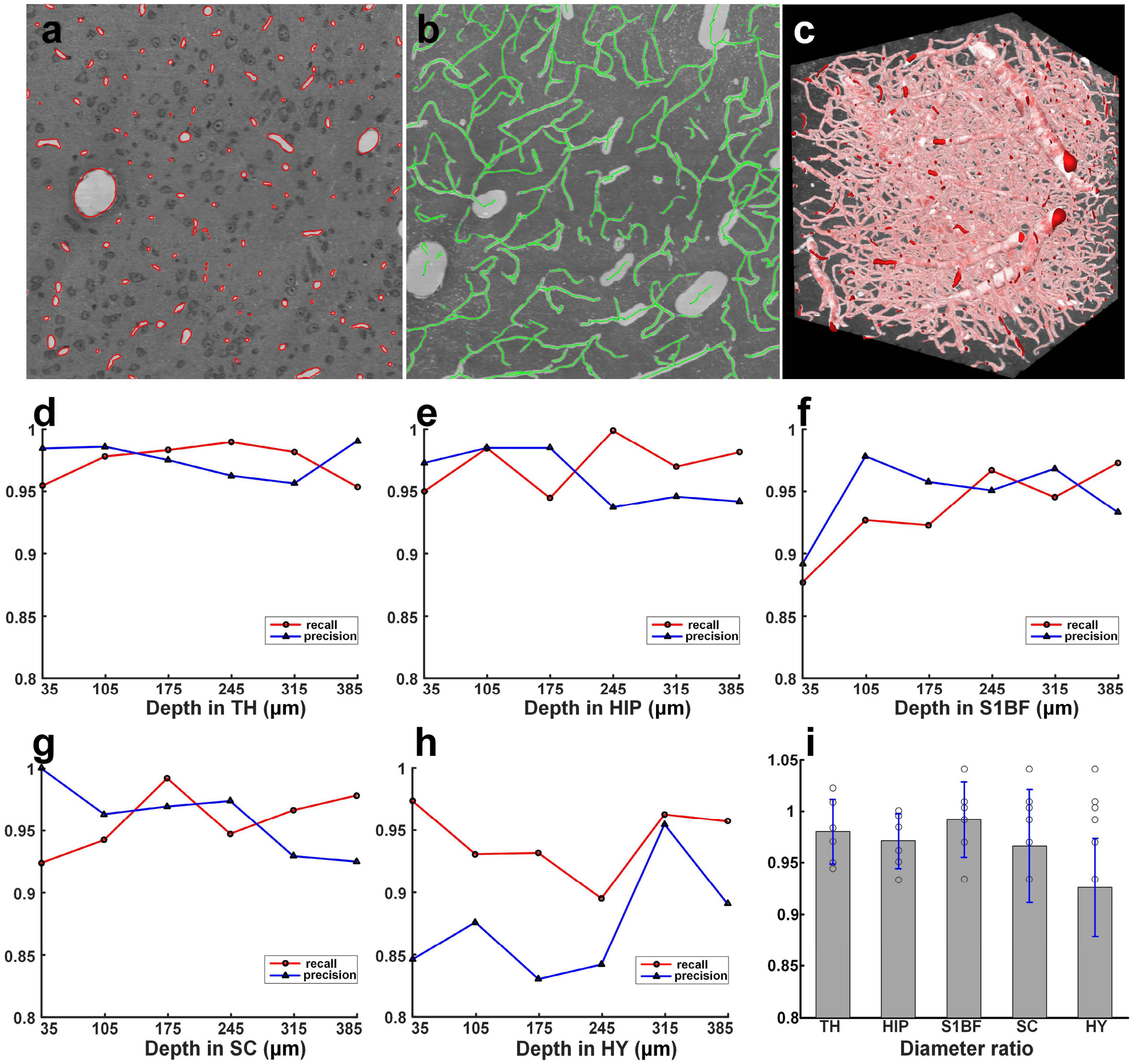

Supplementary Figure 6. Quantitative assessments of the traced microvessels. (a) The represented traced results shown together with the raw data. (b) The centerlines overlapped on the image with thickness of 35 μm. (c) The reconstructed vascular structure together shown together with the raw data in 3D. (d-h) The Recall and Precision results tested between the traced results and manual segmented results of five brain regions(TH, HIP, S1BF, SC, HY). (i) The diameter ratios between the traced results and manual segmented results of the five brain regions. SC = superior colliculus; HIP = hippocampus; TH = thalamus; HY = hypothalamus.

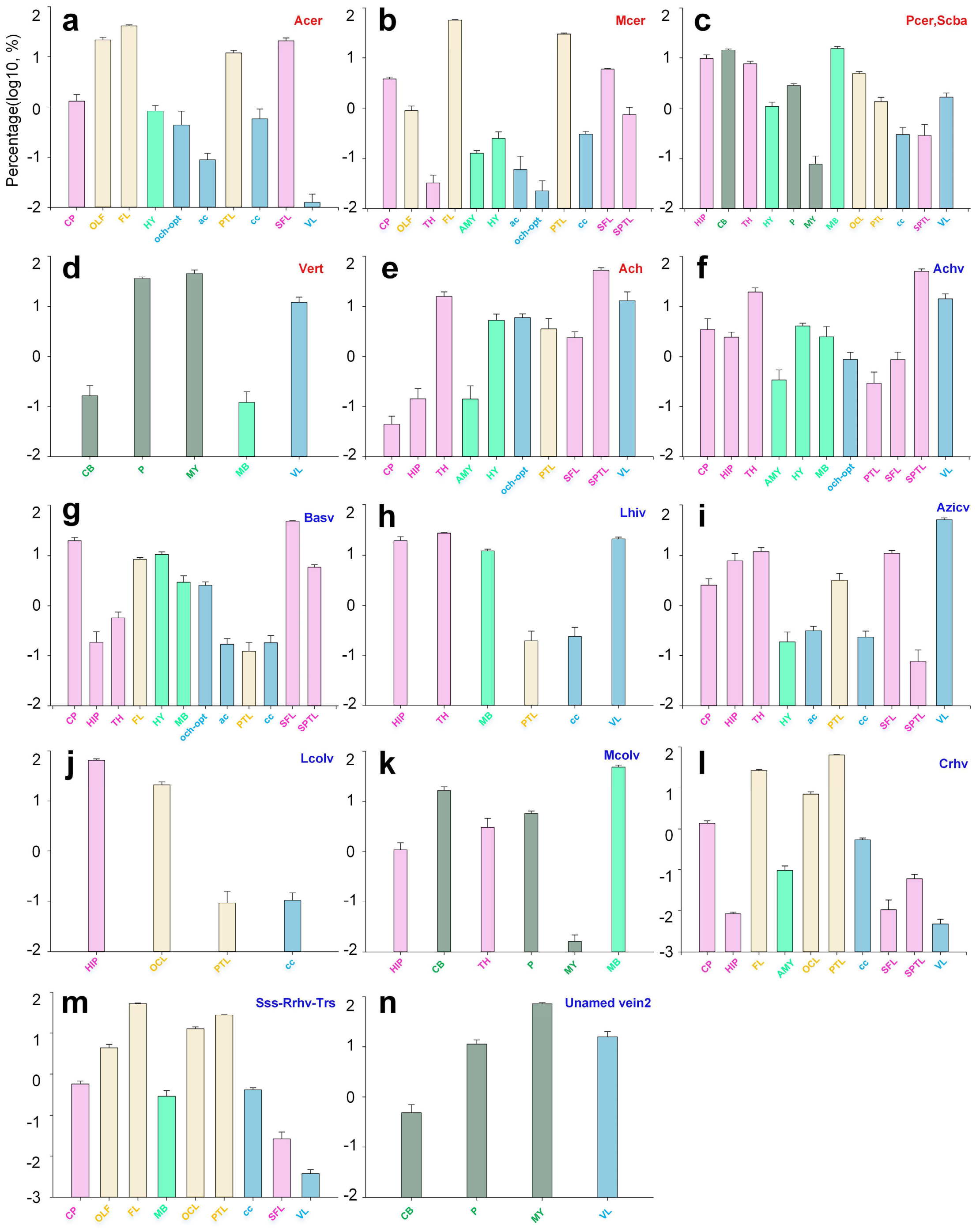

Supplementary Figure 7. Statistical results of the volume-distribution portions of each artery and vein within different brain regions. The segmented brain regions are presented on the horizontal axis, and the distribution portions (after transformation by log10, n=5) of vessels in the corresponding brain region are presented on the vertical axis. (a-e) and (f-n) present the statistical results of the arteries and veins, respectively. A list of abbreviations and the full names of the brain regions is presented in Supplementary Table 1, while a tree of abbreviations and the full names of the vessels is presented in Figure 4.

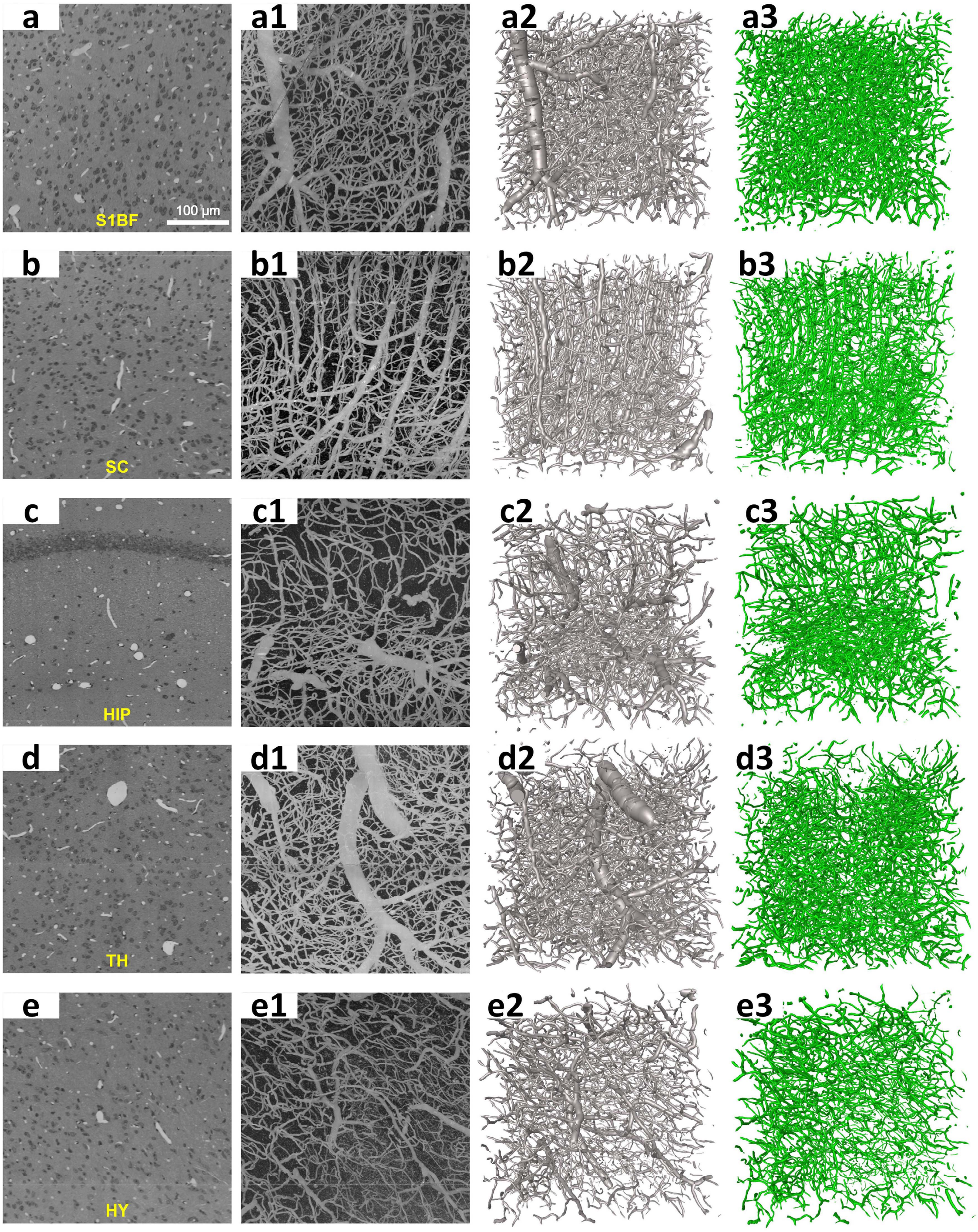

Supplementary Figure 8. Extracted capillary bed in five brain regions. (a-e) Images of the cytoarchitecture and vessels with a thickness of 1 μm in the HIP, SIBF, SC, TH, and HY, respectively (image size of 400 μm x 400 μm). (a1-e1) Maximum projection of 200 μm to show the vessels in the five mouse brains respectively (image size of 400 μm x 400 μm). (a2-e2) Reconstructed fine vascular network in the five mouse brains respectively (stack size of 400 μm x 400 μm x 400 μm). (a3-e3) Separated capillaries in the five mouse brains with diameter size of 8μm (stack size of 400 μm x 400 μm x 400 μm).

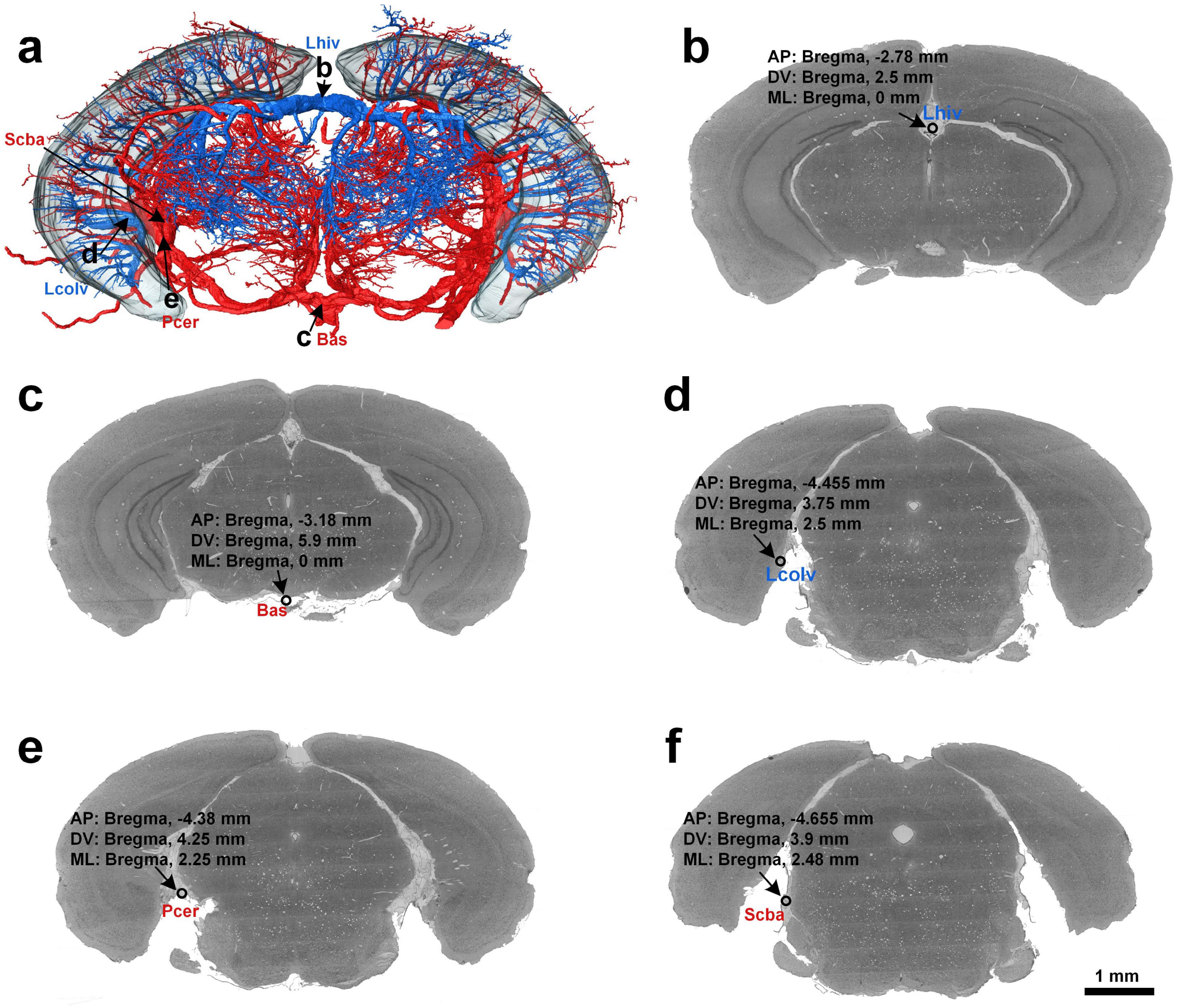

Supplementary Figure 9. Coordinates of specific arteries and veins. (a) 3D structures of the specific arteries and veins, and the locations indicated in b-f. (b) The coordinates of the point where the GCV connects to the Lhiv. (c) The coordinates of the point where Bas connects to the Scba. (d) The coordinates of the point where the Lcolv gives rise to the Vehiv. (e) The coordinates of the point where the Pcer gives rise to Pmch, Lhia and Trcol. (f) The coordinates of the point where the Scba splits into Lscb and Mscb.

1.2 Supplementary Table

Supplementary Table 1: List of abbreviations and the full names of 19 brain regions. The names of the brain regions are arranged in alphabetical order.

Abbreviation	Full name	Abbreviation	Full name
ac	Anterior commissure	OCL	Occipital lobe
AMY	Amygdala	OLF	Olfactory bulb
cc	Corpus callosum	och-opt	Optical chiasm-optic tract
CP	Striatum	P	Pons
CB	Cerebellum	PTL	parietal temporal lobe
FL	Frontal lobe	SFL	Sub- frontal lobe
HY	Hypothalamus	SPTL	Sub-parietal temporal lobe
HIP	Hippocampus	TH	Thalamus
MB	Midbrain	VL	Ventricle
MY	Medulla		

1.3 Supplementary Notes

Ictd:

Anterior cerebral artery (Acer): Branches are ordered from ventral to dorsal. First, the **olfactory artery** (**Olfa**) (series missing in datasets) branches off at the origin of Acer and heads in the rostral to caudal direction on the ventral pial surface and extends to the caudal OLF. The **lateral hypothalamic artery (Lhy)** branches off near the origin of Acer, immediately following the emergence of the Olfa. Lhy heads to the overlying HY in the ventral to dorsal direction. **Azygos of anterior cerebral artery** (**Azac)** originates at the Acer fusion in the ventral aspect of the FL and the caudal aspect of the OLF. Azac runs dorsally and slightly caudally to the dorsal part of the cc. **The lateral orbitofrontal artery (Lofr)** branches off at the ventral Azac, immediately posterior to the origin of Azac. The artery extends medial-dorsal and lateral-dorsal to the OLF and heads in the ventral direction to the caudal dorsal part of the OLF. Lofr gives off two branches, the **lateral branch of the Lofr (Lob)** and the **medial branch of the Lofr (Mob)**. Lob heads dorsally along the lateral surface of the OLF from the ventral direction, while Mob travels through the medial surface of the OLF. The **medial orbitofrontal artery (Mofr)** branches off of the Azac immediately posterior to the Mob near the junction of the OLF and FL. The artery runs dorsal-rostrally and extends to the dorsal portion of the caudal OLF and anterior FL. Mofr gives off two branches: the **olfactory branch (Olo)** and **cortical branch (Cof)**. Olo travels dorsally and a bit rostrally to the caudal-dorsal part of the OLF, while Cof travels in the same path of the Olf to the anterior-dorsal aspect of the FL. The Azac gives off **ascending septal artery (Asa),** which heads in the dorsal direction along the anterior aspect of the septal nuclei to the rostral aspect of the CP and the ventral aspect of the FL. The **rostral septal artery (Rsa)** emerges from the Azac immediately posterior to the origin of the Asa, heading caudally and slightly dorsally toward the rostral region of the HIP. The **anterior internal frontal artery (Aif)** emerges from Azac or the front end of the **azygos pericallosal artery (Azp)**, in the ventral area of the frontal cortex. The artery runs dorsally to the dorsal aspect of the FL. Finally, the Azac forms the Azp, posterior to the Rsa. Azp runs extensively in the direction of the cc, extending to the intersection between the occipital cortex and MB. Then, the Azp gives off the **middle internal frontal artery (Mif)**, which extends dorsally to the dorsal aspect of the PTL. The **posterior internal frontal artery (Pif)** originating from the Azp posterior to the Mif, travels caudally to the OCL and its dorsal surface. The Azp then gives off **retrosplenial artery (Rea)**, which continues in the same path as the Pif to the posterior portion of the brain and the area of the OCL. The **subfornical artery (Sfa)** emerges from the right cerebral branch of the Rea and travels caudal-ventrally to the posterior-ventral aspect of the cc.

Middle cerebral artery (Mcer): The **anterior medial striate artery (Amstr)** branches off first at the ventral aspect of Mcer, extending to the anterior-medial area of the CP. Close to the origin of Mcer, the **lateral hypothalamic artery (Lhy)** is given off in the caudal aspect of the TH, the medial aspect of the CP, and the lateral aspect of the HY. The **piriform artery (Pira)** is given off close to the origin of the Mcer and heads in an anterior direction along the dorsal side to the lateral aspect of the piriform cortex. The **corticostriate artery (**Costr**)** branches off from Mcer following the origin of Pira and heads in a rostral direction along the lateral aspect of the piriform cortex to the caudal aspect of the OLF. The **rhinal artery (Rha)** originates from Mcer, following the origin of Pira, and runs over the surface of the olfactory cortex to the lateral midline. The **anterior striate artery (Astr)** originates from Costr, travelling in the ventral-dorsal direction to the dorsal side of the anterior part of the CP. The **posterior striate arteries (Pstr)** branches off of the main trunk of Mcer near the origin of Costr. The artery travels in the ventral to dorsal direction and extends to the dorsal aspect of

the mid-posterior portion of the CP. The distribution of Pstr is nearly identical to that of Astr. The **rostral branch of Mcer (Ros)** branches off of Mcer around the lateral portion of the midline and heads to the dorsal aspect of the FL via the lateral surface of the FL. The **middle branch of Mcer (Mid)** originates from the dorsal area of Mcer and travels from the anterior portion of the PTL to the OCL. The **caudal branch of Mcer (Caud)** is also given off from the dorsal area of Mcer and form a furcation with the middle branch of Mcer. The artery extends to the dorsal and lateral areas of the PTL to the OCL not covered by Mid.

Posterior cerebral artery (Pcer): The **cortical branch of the Pcer (Copc)** branches off of Pcer and extends to the pial surface through the caudal surface of the OCL and then extends in the caudal to rostral direction on the pial surface to supply the cerebral cortex not covered by Acer and Mcer. Then, Pcer gives off four main arteries at the mid-posterior part of the HIP: the **posterior lateral choroidal artery (Plch)**, **posterior medial choroidal artery (Pmch)**, **longitudinal hippocampal artery (Lhia)** and **transverse collicular artery (Trcol)**. The Plch heads in a rostral direction along the HIP and ends at the posterior and lateral choroid of the lateral VL over the medial HIP and the area above the lateral aspect of the TH. Lhia extends to the top of the dorsal HIP over the medial surface of the HIP, while some Copc branch off and extend to the pial surface. The **transverse hippocampal artery (Trhia)** branches off of Lhia and passes through the HIP from the rostral to caudal direction. The Pmch is almost parallel to the Lhia and extends to the posterior and medial choroid of the lateral VL. The Trcol extends dorsally and caudally to the medial and dorsal surface of the SC. Then, Trcol gives off the **supracollicular arterial network (Scol)**, which covers the dorsal surface of the SC and penetrates into the SC.

Anterior choroidal artery (Acha): The **infundibular artery (Infa)** branches off first from **Ictd** in the ventral surface of the HY. Infa is located on the ventral surface of the brain and heads in a dorsal direction (difficult to distinguish from the Lhy). The **lateral hypothalamic artery (Lhy)** is given off from the Ictd near the origin of Infa and then extends dorsally to the lateral-dorsal aspect of the HY. The **corticoamygdaloid artery (Coamg)** branches off of Ictd next to the origin of Lhy on the ventral surface of the amygdalar area. Coamg gives off small **anterior amygdaloid artery (Ama)**, which penetrates into the AMY. The **anterior choroidal artery (Acha)**, one of the four major arteries that Ictd gives off, originates near the origin of Coamg and extends to the dorsal and medial aspect of the TH and the anterior choroid of the lateral VL. The **posterior amygdaloid artery (Pamy)** branches off of Acha and runs up the lateral side of the AMY to the posterior AMY. As Acha runs, it splits into two major arteries: the **ventral thalamic artery (Vth)** and **dorsal lateral thalamic artery (Dlth)**. The Vth extends to the ventral aspect of the TH and lies on the posterior-ventral aspect of the middle area of the brain, the dorsal HY and the ventral TH. Next to the Vth, the Dlth extends to the middle TH in a slightly dorsal-caudal direction, lying on the mid-posterior portion of the brain and the lateral aspect of the TH.

Vertebral Artery (Vert):

The vertebral artery (Vert) is located on the ventral MY and reaches the anterior portion of the MY and the posterior portion of the P. The **paraolivary artery (Pol)** (lost during specimen preparation, it could only be found in one mouse brain), which originates from Vert and ends at the P, is mainly located on the caudal aspect of the CB and the lateral aspect of the MY. The **basilar artery (Bas)** originates from the fusion of the two Verts and extends to the junction between the MB and P, near the ventral midline of the P and the caudal aspect of the MB. The **median medullary artery (Mmd)** **(**difficult to distinguish) extends into the MY in a dorsal direction along the midline of the ventral MY. The **anterior inferior cerebellar artery** (**Aica**) originates from the Bas around the midpoint of the P and heads in a dorsal direction to split branches into the P and the dorsal aspect into the caudal-

ventral aspect of the CB. The **medial pontine arteries** (**Mpn**), in the medial area of the ventral aspect of the P, branches off of the Bas and travels in a dorsal direction toward the P. At the middle area, ventral to the anterior portion of the P, the **internal auditory artery** (**Iaud**), given off from the Bas, extends to the dorsal aspect of the P and the rostral-ventral aspect of the CB along the dorsal direction. The **medial mesencephalic artery** (**Mmes**) branches off around the caudal area of the Bas, heading into the MB in a dorsal and slightly caudal direction. The Bas extends along the midline in the ventral surface of the P to the most anterior portion of P and the caudal aspect of the TH to form the **superior cerebellar artery** (**Scba**). Scba originates anterior to the Iaud and runs dorsally to the CB and is mainly located on the rostral aspect of the P, the CB and the caudal aspect of the MB. Scba gives off the **posterior communicating artery** (**Pcom**), which is next to Aica. Pcom travels over the ventral surface of MB and connects Pcer with Scba, establishing a relationship between two blood supply sources. The **thalamoperforating artery (Thp)** originates from Scba at the midline of the ventral aspect of the TH and extends to both sides and the dorsal area of the TH. Then, Scba splits into two major branches in the ventral aspect of the anterior CB: the **lateral superior cerebellar arterys** (**Lscb**) and the **medial superior cerebellar artery** (**Mscb**). Lscb travels from the ventral aspect of the anterior CB to the caudal-ventral aspect of the CB over the dorsal aspect of the paraflocculus. Mscb is seen on the dorsal aspect of the MB and the rostral-dorsal aspect of the CB and gives off the **dorsal cerebellar artery** (**Dcb**) and **dorsomedial cerebellar artery** (**Dmcb**). Dcb runs dorsally to the dorsal aspect of the CB and are located on the dorsal aspect of the MB and CB. Dmcb extends to the ventral-caudal aspect of the CB.

Veins:

Dorsal Veins (Dov)

The **rostral rhinal vein** (**Rrhv**) stems from the rostral confluence of the sinus and extends dorsal-ventrally to the ventral aspect of the FL. The **dorsal frontal branch (Dfl)**, **lateral frontal branch** (**Lfl**) and **ventral frontal branch** (**Vfl**) are the three surficial branches that branch off from the main trunk of the Rrhv and extend to different aspects of the FL. Dfl travels in a ventral and slightly caudal direction to the dorsal surface of the FL. Lfl heads in the same direction as Dfl and extends to the lateral surface of the FL. Vfl runs ventral-caudally to the ventral FL. All three branches give off **frontal lobe penetrating veins (Flp)**, which penetrate into areas of the FL and SFL.

The **superior sagittal sinus (Sss)** emerges from the rostral confluence of the sinus and travels rostral-caudally from the caudal aspect of the OLF to the point where the occipital cortex meets the SC. Sss is mainly located on the midline and dorsal aspect of the brain. The Sss divides into three surficial branches: **rostral branch of the superior sagittal sinus (Ross)**, **middle branch of the superior sagittal sinus (Mids)** and **caudal branch of the superior sagittal sinus** (**Cauds**). Ross heads ventrally and slightly caudally and extend to the dorsal aspect of the FL and PTL. Mids lies in the middle portion of the brain and the dorsal aspect of the PTL, traveling in the same direction as Ross to the dorsal aspect of the PTL. Cauds head ventrally and slightly rostrally, extending to the dorsal aspect of the PTL and the OCL. All three branches give off **cortical penetrating veins (Cops)**, which penetrate into areas of the cortex and sub-cortex.

The **transverse sinus** (**Trs**) originates from the caudal confluence of the sinus and travels dorsal-ventrally to the emergence of the **caudal rhinal vein** (**Crhv**) before extending to the lateral brain. Trs lies on the junction between the OCL and the CB. The **occipital cortical vein (Occ)**

branches off of the trunk of the Trs and travels in a rostral-ventral direction from the caudal brain to the OCL.

The **caudal rhinal vein** (**Crhv**) arising from the Trs travels rostrally along the lateral midline into the caudal region of the FL via the rhinal cortex. There are three dorsal branches arising from the trunk of the Crhv. The **dorsal rostral branch of Crhv (Dorc)**, traveling rostrally and slightly dorsally, extends to the lateral and dorsal aspect of the PTL and the lateral and caudal part of the FL. The **dorsal middle branch of Crhv (Domc)** runs in a dorsal-rostral direction to reach the lateral and dorsal aspect of the PTL. The **dorsal caudal branch of Crhv (Doccs)** extends dorsally to the OCL and the lateral-dorsal area of the PTL. Tree branches also emerge ventrally from the trunk of the Crhv. The **ventral rostral branch of Crhv** (**Verc**) travels caudal-ventrally to the piriform cortex. The **ventral middle branch of Crhv** (**Vemc**) travels in the same direction as Verc and extends to the lateral region of the rhinal cortex. The **ventral caudal branch of Crhv** (**Vecc**) travels ventrally and slightly rostrally to the lateral aspect of the AMY. All six branches give off **cortical penetrating veins (Cops)**, which penetrate into areas of the cortex and sub-cortex.

Deep Veins(Dov)

The **vein of Galen (Gcv)** originating from the **straight sinus** (**sts**) connects to the caudal confluence of the sinus and penetrates into the brain in the area where the occipital cortex meets the SC. The **longitudinal hippocampal vein** (**Lhiv)** is connected to Gcv, and there are two major veins that branch off from **Lhiv**, including the **ventral hippocampal vein** (**Vehiv**) and **dorsal thalamic vein** (**Dthv**). Vehiv travels to the middle area of the ventral HIP from the ventral aspect of the HIP and the dorsal aspect of the TH. Vehiv gives off the **transverse hippocampal vein** (**Trhiv**), which runs dorsal-rostrally to pass through the HIP from the caudal to rostral area in the upper region of the HIP. Dthv travels ventral-laterally to the lateral aspect of the TH. **thalamoperforating vein** (**Thpv**) originated from Dthv, penetrates into the TH in the rostral and lateral areas, mainly lying in the dorsal aspect of the TH, and Thpv also gives off Trhiv.

The **azygos internal cerebral vein** (**Azicv**) also originates from Gcv, anterior to the origin of Lhiv. The **dorsal septal vein** (**Dsv**) branches off of Azicv and travels caudal-ventrally to the dorsal aspect of the septal nucleus and medial aspect of the two CP. Then the Azicv gives off **thalamostriate vein** (**Thsv**) and the **lateral choroidal vein** (**Lchv**). Thsv heads in a rostral and ventral direction to the medial aspect of the CP, while the terminal branches curve around in a caudal and ventral direction to the rostral aspect of the TH. Lchv extends to the lateral aspect of the choroidal vascular network in a lateral direction over the VL between the CP and the HIP.

The **lateral collicular vein (Lcolv)** originates from the Trs, reaching the lower portion of the HIP. The Lcolv gives rise to the **ventral transverse hippocampal vein (Vehiv),** which penetrates the HIP via the dorsal part and reach the rostral part, surrounding the lower portion of the HIP, and the **cortical branch vein (Corv)** connects Lcolv and Trs in the caudal area of the OCL.

The **medial collicular vein** (**Mcolv**) is located in the caudal aspect of the brain, giving off the **rostral ventral cerebellar vein** (**Rovc**) at the dorsal aspect of the SC. Rovc heads ventrally, eventually contacting the CB and MB. Then, Rovc splits into two branches, the **rostral cerebellar branch** (**Roce**) and **caudal mesencephalic branch** (**Came**), which extend into the CB and MB, respectively. Came heads into the MB in the rostral direction and surrounds the rostral-ventral aspect of the MB. At the lateral aspect of the MB, the **lateral mesencephalic branch** (**Lame**) branches off from Rovc and extends into the MB over the lateral surface of the MB. The **lateral thalamic branch** (**Lath**) given off from Rovc travels along the lateral surface of the TH to its interior and rostral aspect.

If **Vthv** of **Achv** exists, **Lath** will not exist, whereas if Vthv is missing, Lath will exist; thus, **Lath** can also be called **Vthv**.

Ventral Veins (Vev)

The **basal vein (Basv)** first gives rise to two major veins, the **anterior cerebral vein (Acerv)** and the **middle cerebral vein (Mcerv)**, which are almost parallel to Acer and Mcer, respectively, but the extending distance to the dorsal area is short. As Mcerv extends, the **rhinal vein (Rhv)** and **piriform vein (Pirv)** are given off on the ventral pial surface. Rhv extends dorsally and caudally exactly like Rha mentioned above, while **Pira** extends anteriorly and slightly dorsally to the piriform cortex. Then, the **lateral hypothalamic vein** (**Lhyv**) and **infundibular vein** (**Infv**) branch off. **Infv** is difficult to distinguish from Lhyv; both head dorsally from the ventral surface to the HY. Then, the **anterior choroidal vein** (**Achv**) is formed; the **lateral hypothalamic vein** (**Lhyv**) and **corticoamygdaloid veins** (**Coamgv**) are also formed near the origin of Achv. The extending patterns of Lhyv is almost the same with the Lhyv described above. Coamgv travels on the ventral surface of the AMY similar to Coamg mentioned above. Achv is almost parallel to Ach mentioned above, which also extends to the dorsal and medial aspect of the TH, the anterior choroid of the lateral VL, and more to the caudal CP. The **posterior amygdaloid veins (Pamv)** branches off from Achv and runs dorsally and caudally through the lateral side of the AMY to the posterior AMY. The **ventral thalamic vein (Vthv)** branches off from Achv and extends dorsally first and then returns with a flat curve to extend ventrally and medially to the ventral aspect of the TH. Next to Vthv, the **dorsal lateral thalamic vein (Dlthv)** originated from Achv, extends in exactly the same direction as Vthv to the middle TH. The **caudal striatum vein (Cstrv)** branches off from Achv at the rostral sides of Dlthv, extending into the medial region of the caudal part of the CP.

Caudal Veins (Cvv)

The **Unamedv** contains **Unamedv1 (Un1)** and **Unamedv2 (Un2)**. **Un1** first extends caudally on the dorsal surface of the CB and then penetrates into the CB almost perpendicularly. **Un2** is located on the dorsal surface of the P and extends rostrally and laterally at the area between the CB and P before penetrating the P and generating many branches.
